# Therapeutic Potential of Lipid Nanoparticle‐Encapsulated CD19‐Targeting mRNAs in Lupus and Rheumatoid Arthritis

**DOI:** 10.1002/advs.202501628

**Published:** 2025-04-07

**Authors:** Chipeng Guo, Yingsen Tang, Ling Zeng, Xiaomei You, Siweier Luo, Yufei Du, Le Wang, Liangchun Wang, Jianchuan Wang, Jinjin Chen, Yiming Zhou

**Affiliations:** ^1^ Department of Dermatology Sun Yat‐sen Memorial Hospital Sun Yat‐sen University Guangzhou 510120 China; ^2^ Basic and Translational Medical Research Center Sun Yat‐sen Memorial Hospital Sun Yat‐sen University Guangzhou 510120 China; ^3^ School of Pharmaceutical Sciences Sun Yat‐sen University Guangzhou 510006 China

**Keywords:** CD19, lupus, mRNA‐LNPs, rheumatoid arthritis

## Abstract

The hyperactivation of autoreactive B cells and plasma cells leads to the development of systemic lupus erythematosus (SLE) and rheumatoid arthritis (RA), therefore, targeting the abnormal B cells and plasma cells might hold promise for the treatment of these refractory and relapsing diseases. This study developed **l**ipid **n**ano**p**article‐encapsulated **mRN**A‐encoding **a**nti**b**odies (mRNab‐LNPs) targeting CD19, and evaluated their therapeutic efficacy in lupus and RA mice. mRNab‐LNPs enabled robust production of anti‐CD19 antibodies in multiple cell lines in vitro. Interestingly, intramuscular injection of mRNab‐LNPs resulted in high and sustained production of anti‐CD19 antibodies in mice. In particular, the numbers of CD19+ circulating B cells and tissue‐resident plasma cells are significantly reduced by mRNab‐LNPs in mice. As a result, mRNab‐LNPs significantly reduced the histopathological changes and tissue injuries in both lupus and RA mice. Collectively, these findings demonstrate the therapeutic and translational potential of mRNab‐LNPs in the treatment of SLE and RA.

## Graphical abstract

This study develops lipid nanoparticle‐encapsulated mRNA‐encoding antibodies (mRNab‐LNPs) targeting CD19, demonstrating robust anti‐CD19 antibody production in vivo. Intramuscular injection of mRNab‐LNPs reduces CD19+ B and plasma cells in lupus and rheumatoid arthritis mice, significantly alleviating histopathological changes and tissue injuries of skins, kidneys, and joints. These findings highlight the therapeutic and translational potential of mRNab‐LNPs for treating refractory autoimmune diseases.

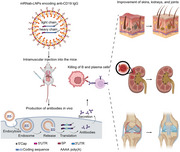

## 1 Introduction

Systemic lupus erythematosus (SLE) and rheumatoid arthritis (RA) are two major autoimmunity diseases with multi‐organ involvement that appear to have an alternate relapsing and remitting course and are associated with high rates of disability and mortality in patients.^[^ ^1^ ^]^ The hyperactivation of autoreactive B cells and plasma cells plays a crucial role in the pathogenesis of SLE and RA,^[^ ^2^ ^]^ therefore, there is an urgent need to profoundly reset the abnormal humoral immune system to achieve drug‐free remission.^[^ ^1^ ^]^ To date, various anti‐B cell drugs and agents have been indicated for the treatment of SLE and RA.^[^ ^2^, ^3^ ^]^ However, the clinical efficacy has not always been satisfactory.^[^ ^4^ ^]^ The previous CD20‐targeting therapies fail to achieve the desired clinical endpoints, in part because of their inability to deplete the short‐lived and long‐lived plasma cells.^[^ ^4^, ^5^ ^]^ Besides, though belimumab, a monoclonal antibody targeting B‐cell activating factor (BAFF), was the first approved therapeutic agent for SLE, plasma cells can still be sustained by a proliferation‐inducing ligand in the absence of BAFF.^[^ ^6^ ^]^ In addition, the CD20‐negative plasma cells might lead to treatment resistance in RA.^[^ ^7^ ^]^ As a result, specific therapeutic targets for more complete depletion of B cells and plasma cells are of clinical importance.

CD19 is widely expressed from pro‐B cells to memory B cells and short‐lived plasma cells, making it a target for B cell and plasma cell depletion therapies.^[^ ^8^ ^]^ Initially, anti‐CD19 antibodies was used for the hematologic malignancies, which was designed to induce tumor cell lysis.^[^ ^9^ ^]^ More recently, chimeric antigen receptor (CAR)‐T cell therapy has been developed for multiple types of tumors and autoimmunity diseases.^[^ ^10^ ^]^ Notably, anti‐CD19 CAR‐T cell therapy has shown high efficacy in refractory SLE patients,^[^ ^11^ ^]^ suggesting that CD19 may be a potential target for complete depletion of B cells and plasma cells, and resetting of the humoral immunity in SLE patients. However, the requirement for pre‐lymphodepletion and the associated toxicities such as fatal cytokine release syndrome^[^ ^3^ ^]^ might limit the clinical application. In addition, patients receiving autologous CAR‐T cell immunotherapies are at potential risks for T cell malignancies.^[^ ^12^ ^]^ Therefore, development of an alternative therapy targeting CD19 to deplete autoreactive B and plasma cells is of significance.

Lipid nanoparticle‐encapsulated messenger RNA (mRNA‐LNPs) has recently underlined success in the development of COVID‐19 vaccines,^[^ ^13^ ^]^ and has shown promising results against tumors and autoimmune diseases, such as myasthenia gravis.^[^ ^14^ ^]^ Different therapies based on mRNA have shown well‐tolerated safety and enables the rapid expression of proteins and antibodies in vivo.^[^ ^15^ ^]^ In addition, the simplified manufacturing processes and the lower production cost make it more feasible for clinical applications.^[^ ^16^ ^]^ After infusion, the mRNA‐LNPs are mainly endocytosed by hepatocytes and translated into new proteins in the hepatocytes, and in this way, individuals who have received mRNA‐LNPs can stably produce therapeutic proteins in one's own body.^[^ ^17^ ^]^ In this study, we have developed LNPs‐encapsulated **mRN**A‐encoding **a**nti**b**ody (mRNab‐LNPs) that enables a high and sustained production of anti‐mouse CD19 (anti‐mCD19) antibodies targeting autoreactive B cells and plasma cells simultaneously. Treatment with mRNab‐LNPs enables robust production of anti‐CD19 antibodies in multiple cell lines in vitro. Interestingly, intramuscular injection of mRNab‐LNPs resulted in high and sustained production of anti‐CD19 antibodies in mice. In particular, the numbers of CD19+ circulating B cells and tissue‐resident CD19+CD138+ plasma cells were significantly reduced by mRNab‐LNPs in MRL/lpr lupus and RA mice. In conclusion, our data highlight the therapeutic and translational potential of mRNab‐LNPs targeting CD19 for autoreactive B‐ and plasma cell‐meditated autoimmune diseases.

## 2 Results

### 2.1 Construction and In Vitro Verification of the Lipid Nanoparticle‐Encapsulated mRNA‐Encoding Anti‐Mouse CD19 Antibodies (mRNab‐LNPs)

**Figure 1:**
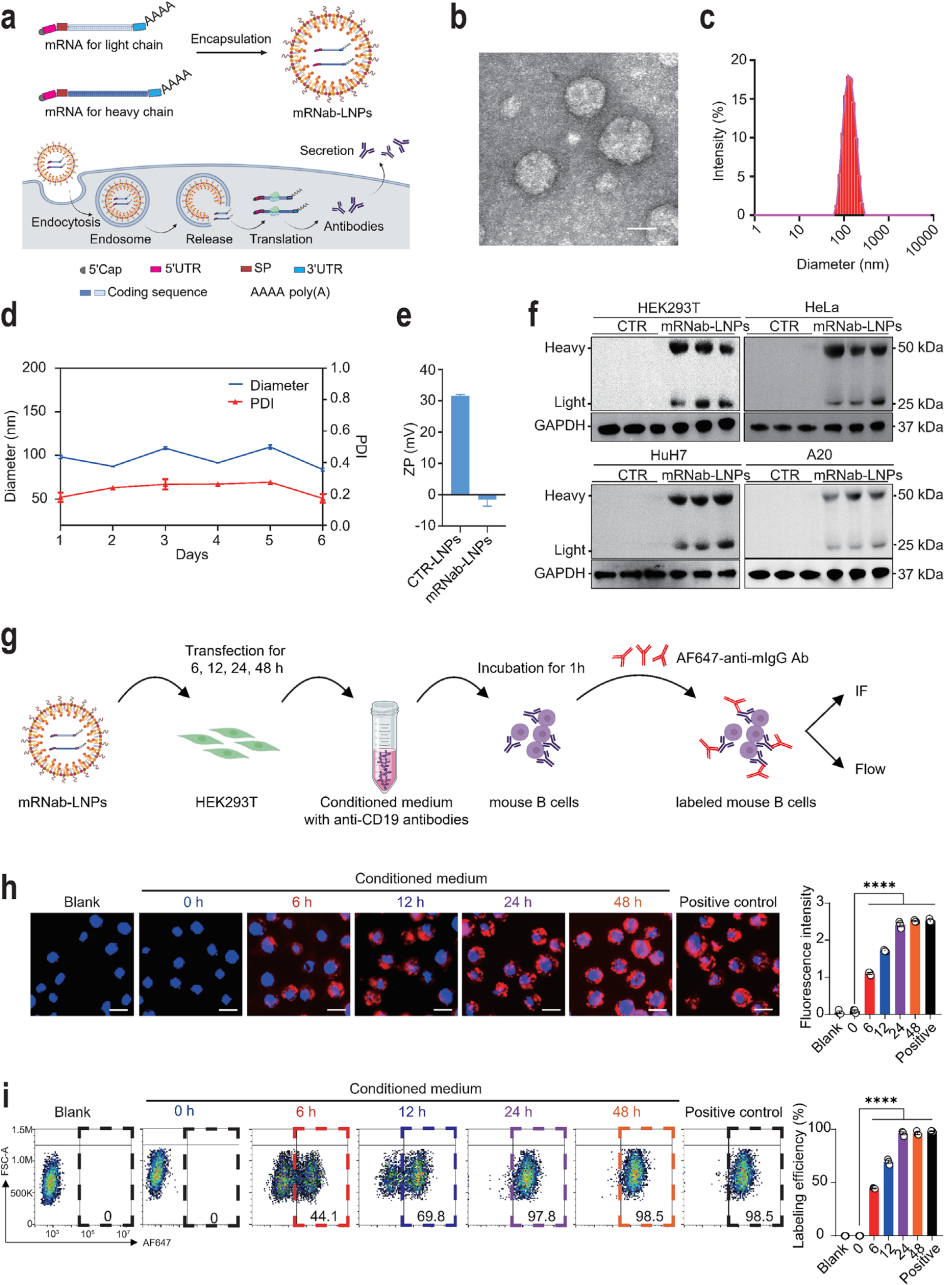
Construction and in vitro verification of mRNab‐LNPs. a) Schematic diagram showing the construction of lipid nanoparticles‐encapsulated mRNA‐encoding anti‐mouse CD19 antibodies (mRNab‐LNPs), and the antibody generation by mRNab‐LNPs in cells. b) The TEM images of the mRNab‐LNPs. c) Biophysical characterization of the mRNab‐LNPs by Dynamic light scattering (DLS) measurement. d) Size analysis and polydispersity index values (PDIs) for mRNab‐LNPs stored at 4 °C within 7 days. e) The zeta potential (ZP) of empty LNPs (CTR‐LNPs) and mRNab‐LNPs. f) Western blots showing the expression of the mRNA‐encoding heavy and light chains of the anti‐mouse CD19 IgG from the cell lysis of HEK293T, HeLa, HuH7, and A20 cells transfected with empty LNPs and mRNab‐LNPs, respectively (*n* = 3 each). g) Schematic diagram of the experimental design for the detection of the secreted anti‐mCD19 antibodies in vitro. HEK293T cells were transfected with mRNab‐LNPs (400 ng) for 6, 12, 24, and 48 h, respectively. Then, the conditioned media with anti‐CD19 antibodies of the transfected HEK293T cells were harvested and added to a mouse B cell line, A20 cells for 1 h. These anti‐CD19 antibody‐labeled A20 cells were then incubated with a secondary antibody (AF647‐conjugated anti‐mouse IgG antibody). Immunofluorescence and flow cytometry analysis were used to quantify the anti‐CD19 antibody levels in A20 cells. Untreated and a commercial AF647‐conjugated anti‐mouse CD19 antibody (100 µg mL^−1^)‐treated A20 cells were used as blank control and positive control, respectively. h) Representative immunofluorescence images of mouse B cells treated with the conditioned media from mRNab‐LNPs transfected HEK293T cells. Fluorescence intensity of CD19 labeling efficiencies for mRNA‐encoding antibodies and conventional antibodies in mouse B cells (*n* = 3 each). i) Representative flow cytometry data of mouse B cells treated with the conditioned media from mRNab‐LNPs transfected HEK293T cells. Percentages of CD19 labeling efficiencies for mRNA‐encoding antibodies and conventional antibodies in mouse B cells (*n* = 3 each). Data are mean ± standard error of the mean. Scale bars = 50 µm. ****p < 0.0001.

According to previous studies,^[^ ^18^ ^]^ the synthetic mRNAs consisted of the 5′ cap, 5′ untranslated region (UTR), signal peptide (SP), protein‐coding sequence, 3′ UTR, and 3′ poly(A) tail. Two mRNAs were designed to separately encode the heavy and light chains of mouse IgG against mouse CD19 (**Figure** **1**a). To construct the mRNab‐LNPs, both heavy chain and light chain‐encoding mRNAs in aqueous solution and lipids in ethanol were mixed to yield self‐assembled mRNab‐LNPs. Detailed procedures are described in the Experimental Section. The structures of the formulated mRNab‐LNPs were visualized by the transmission electron microscopy (TEM), which exhibited solid spheres with homogeneous morphologies (Figure 1b). Dynamic light scattering (DLS) measurements indicated a narrow size distribution of mRNab‐LNPs, with an average diameter of ≈100 nm (Figure 1c). The variations in size and polydispersity index (PDI) were used to evaluate the stability of mRNab‐LNPs stored at 4 °C within 1 week (Figure 1d). The results showed that the size of mRNab‐LNPs remained stable over different time points, with no significant changes in hydrodynamic diameter. In addition, the PDIs showed that the mRNab‐LNPs maintained their structural integrity without aggregation or degradation over time (Figure 1d). The zeta potential (ZP) of LNPs was positive due to the positive charges of cationic lipids.^[^ ^13b^ ^]^ Interestingly, encapsulation of mRNAs dramatically reduced the ZP of LNPs, since the negatively charged mRNA neutralized the positive charge of the LNPs. This change in surface charge indicates that the mRNAs were successfully encapsulated within the LNPs (Figure 1e).

To investigate the cell type selectivity of mRNab‐LNPs, mRNab‐LNPs were then incubated with different cell lines, including HEK293T, HeLa, HuH7, and A20. Interestingly, we found that the mRNab‐LNPs could be endocytosed by different cell lines and encoded the heavy and light chains of the anti‐mCD19 antibodies in all cell lines. These results indicate that we have successfully constructed the mRNab‐LNPs that could produce the anti‐mCD19 antibodies in both human and mouse cells (Figure 1f). To investigate antibody glycosylation, a critical factor in secondary antibody recognition and functionality,^[^ ^19^ ^]^ conventional and mRNA‐encoded antibodies were treated with PNGase F to remove N‐glycans from the heavy chains. Following treatment, a shift in the mobility of the IgG heavy chain was observed in both types of antibodies during electrophoresis, with the protein band size becoming smaller (Figure S1, Supporting Information). This finding demonstrated that the mRNA‐encoded antibody undergoes proper post‐translational modifications, including glycosylation, which are essential for maintaining structural integrity and recognition by secondary antibodies. Additionally, the de‐glycosylation results revealed that the heavy‐to‐light chain ratio for both types of antibodies was ≈1:1, indicating that the mRNA‐encoded antibody achieves an equal production of heavy and light chains (Figure S1, Supporting Information). This further validates the capability of mRNA‐LNP technology to produce antibodies with functional and structural properties equivalent to those of conventional antibodies. To further investigate the antibody generation efficiency of mRNab‐LNPs in vitro, we developed an indirect staining method by measuring the labeling efficiency of the mRNA‐encoding anti‐mCD19 antibodies on A20 cells, a mouse B cell line that expresses CD19 but not mouse IgG. Briefly, the A20 cells were incubated with the conditioned media from HEK293T cells transfected with mRNab‐LNPs for different times (6, 12, 24, and 48 h). Conditioned media from untransfected HKE293T cells was used as a control (0 h). After being labeled with the conditioned media containing mRNA‐encoding anti‐mCD19 antibodies for 1 h, A20 cells were then stained with the AF647‐conjugated anti‐mouse IgG secondary antibodies for 15 min and analyzed by immunofluorescence staining and flow cytometry (Figure 1g). Both the IF staining and flow cytometry results demonstrated that A20 cells treated with the conditioned media of transfection for 6 h showed strong AF647 signals, with a labeling efficiency of 44.1% by flow cytometry. Notably, the labeling efficiency of the conditioned media from HEK293T cells transfected with mRNab‐LNPs for 24 and 48 h reached to 97.8% and 98.5%, respectively, which were similarly with the result from A20 cells treated with conventional anti‐mCD19 antibodies, of which the labeling efficiency was 98.5% (Figure 1h,i). To further calculate the concentrations of mRNA‐encoding antibodies, we first generated a standard dose‐response curve based on the labeling efficiency on the equal number of B cells using the conventional anti‐mCD19 antibodies by flow cytometry, with a EC50 ≈0.35 µg mL^−1^ (Figure S2a, Supporting Information). The antibody concentrations from conditioned media of HEK293T cells transfected with mRNab‐LNPs for different times were calculated based on the standard dose‐response curve from Figure S2a (Supporting Information). The analysis demonstrated a significant increase in antibody concentrations following 6 h of transfection, with peak levels in the supernatant reaching 100 µg mL^−1^ at both 24‐ and 48‐h post‐transfection (Figure S2b, Supporting Information). To assess the quality of the produced antibody, we evaluated its specificity by flow cytometry. Briefly, mouse splenocytes were initially treated with an Fc receptor blocker at 4 °C for 5 min. After washing, the cells were incubated at 37 °C for 1 h with two groups of conditioned media obtained from HEK293T cells transfected with mRNA‐LNPs for 0 and 48 h. Splenocytes expressing the CD19 antigen were able to bind to the mRNA‐encoded anti‐mCD19 antibody, which was subsequently detected by anti‐mouse IgG secondary antibody. The specificity of the mRNA‐encoded antibody was determined by analyzing the percentages of AF647‐anti‐mouse IgG staining across different cell subsets. Flow cytometry analysis revealed that nearly all B220+ B cells were bound by AF647‐anti‐mouse IgG after incubated with conventional anti‐mCD19 antibodies and conditioned media from mRNab‐LNPs‐transfected HEK293T cells. In contrast, CD3+ T cells, Ly6C+ monocytes, and CD56+ NK cells showed less or scarce binding with AF647‐anti‐mouse IgG (Figure S3, Supporting Information). These findings confirm that the mRNA‐encoded antibody exhibits high specificity for CD19 antigen. Taken together, these results suggest that we have successfully constructed the mRNab‐LNPs, which can efficiently generate high‐affinity and high‐specificity anti‐mCD19 antibodies in both human and mouse cells in vitro.

### 2.2 mRNab‐LNPs Showed Advantages in Antibody Production and Pharmacokinetics In Vivo

**Figure 2:**
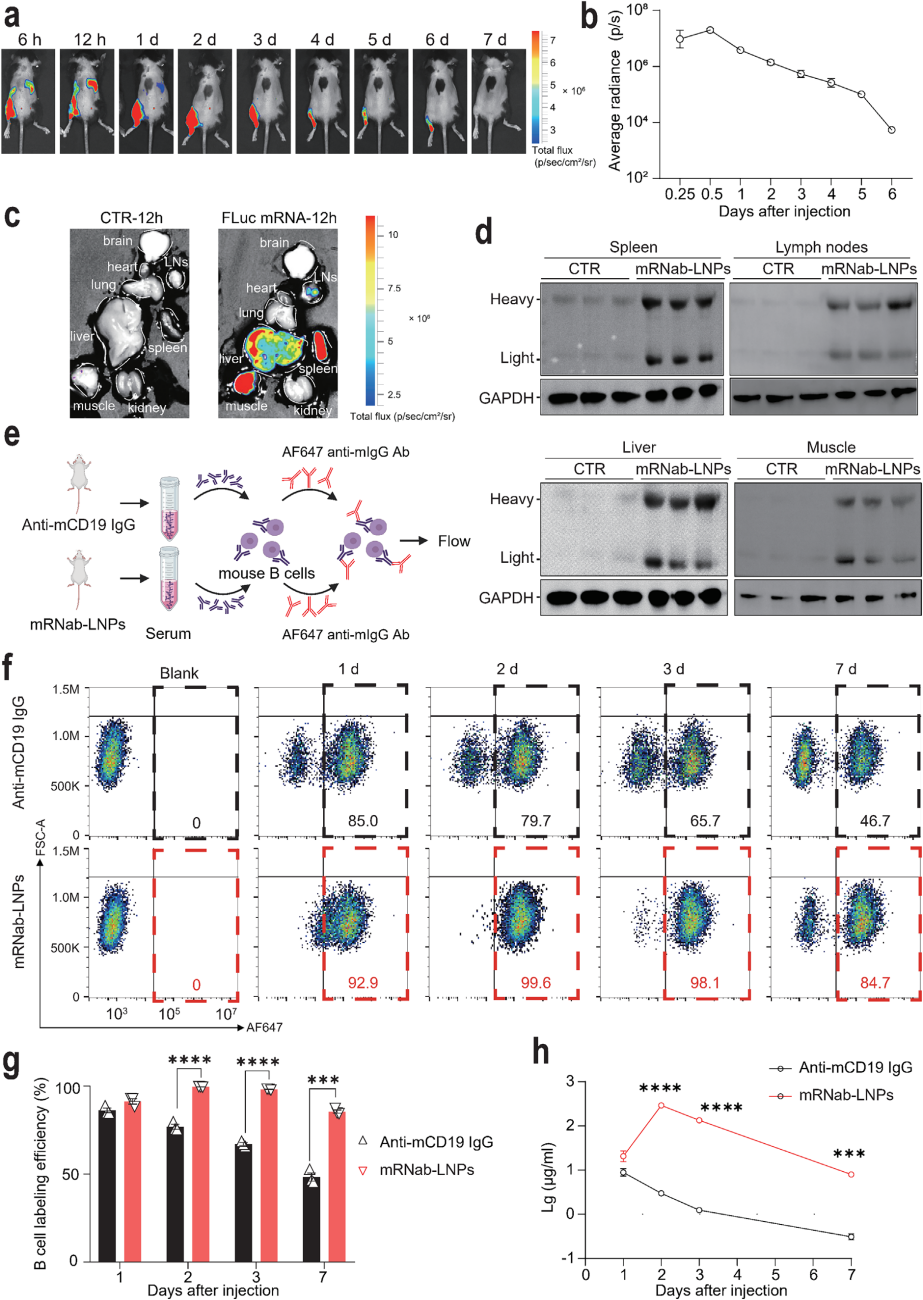
Pharmacodynamics and antibody generation of mRNab‐LNPs in vivo. a) The time‐lapse IVIS spectrum images showing the luciferase signals in C57BL/6 mice injected intramuscularly with the firefly luciferase mRNA (FLuc mRNA)‐LNPs (2.0ug). b) Quantitative results of the Fluc mRNA‐LNPs signals from (a) (*n* = 3 each). c) IVIS Spectrum images showing the tissue distribution of FLuc mRNA‐LNPs in C57BL/6 mice 12 h after the intramuscular injection. LNs, lymph nodes. d) Western blots showing the expression of the mRNA‐encoding mouse IgG heavy and the light chains from spleen, lymph nodes, liver, and muscle of C57BL/6 mice 12 h after the intramuscular injection of empty LNPs or mRNab‐LNPs (1mg kg^−1^), respectively (*n* = 3 each). e) Schematic diagram of the experimental design for the quantification of serum mRNA‐encoding antibodies and conventional antibodies ex vivo. C57BL/6 mice received a single injection of mRNab‐LNPs (1 mg kg^−1^) or anti‐mCD19 antibodies (2.5 mg kg^−1^), respectively. Serums at indicated time points after the injection were harvested and added to mouse B cells for 1 h. The labeled mouse B cells were then labeled with a secondary antibody (AF647‐conjugated anti‐mouse IgG antibodies) for flow cytometry analysis. f) Representative flow cytometry data of mouse B cells treated with serums of mice that were injected with mRNab‐LNPs or anti‐mCD19 antibodies (*n* = 3 each). g) Quantitative results of the percentage of labeled mouse B cell using the mouse serums collected on day 1, 2, 3, and 7 after the injection of mRNab‐LNPs or CD19 antibodies (*n* = 3 each). h) The calculated serum concentrations of anti‐mCD19 antibodies from two groups of mice using the standard curve from Figure S2a (*n* = 3 each). Data are mean ± standard error of the mean. ***p < 0.001, ****p < 0.0001.

To investigate the biodistribution and pharmacokinetics of mRNab‐LNPs in vivo, we constructed the LNPs‐encapsulated mRNA‐encoding firefly luciferase (FLuc mRNA‐LNPs), and injected them intramuscularly to C57BL/6 mice. In Vivo Imaging Systems (IVIS) spectrum results showed that strong bioluminescence signals were detected at the injection site and in the upper abdomen area 6 h after the injection. The signals in the upper abdomen area reached to a maximum level 12 h after the injection. The total bioluminescence signals remained detectable six days after the single injection, during which time the mice were vital and showed no abnormal behavior (**Figure** **2**a,b). Ex vivo imaging analysis further demonstrated the enrichment of bioluminescent signals in the spleen, lymph nodes (LNs), liver, and muscle tissues 12 h after the injection (Figure 2c). Furthermore, Western blotting showed that a single injection of mRNab‐LNPs, but not empty LNPs, resulted in robust production of anti‐mouse CD19 antibodies in spleen, lymph nodes, liver, and muscle tissues after 12 h (Figure 2d). Compared to PBS treatment, LNPs did not affect the body weights, hepatic (AST and ALT), and renal functions (BUN and creatinine) of C57BL/6 mice one week after the injection (Figure S4a,b, Supporting Information). In addition, H&E staining results showed that LNPs did not induce the histological changes in heart, liver, lung, spleen, and kidney of C57BL/6 mice (Figure S4c, Supporting Information). To further examine the early systemic inflammatory response following treatment of mRNab‐LNPs in a diseased context, we measured the serum levels of inflammatory cytokines, including TNF‐α, IL‐1β, and IL‐6, and at 72 h post‐treatment with mRNab‐LNPs in MRL/lpr lupus mice. Interestingly, the results demonstrated that the levels of TNF‐α, IL‐1β, and IL‐6 were comparable between the two groups, indicating that mRNab‐LNP treatment does not induce a lasting systemic inflammatory response (Figure S4d, Supporting Information).

To further evaluate the concentrations and pharmacokinetics in vivo, serum samples from the mice injected intravenously with the conventional anti‐mCD19 antibodies and from the mice injected intramuscularly with mRNab‐LNPs were collected at different time points after the injection (Day 1, 2, 3, and 7). These serum samples were incubated with the A20 cells for 1 h. The labeled A20 cells were then stained with the AF647‐conjugated anti‐mouse IgG secondary antibodies and analyzed using flow cytometry (Figure 2f). The flow cytometry results showed that, although both conventional antibody and mRNab‐LNPs treatment resulted in high labeling efficiencies on mouse B cells (≈85% and ≈92.9%, respectively) 1 day after the injection, the labeling efficiency of conventional antibody decreased more rapidly compared to that of mRNab‐LNPs. In particular, the labeling efficiency of conventional antibody was significantly lower than that of mRNab‐LNPs on day 7 (≈46.7% and ≈84.7%, respectively) (Figure 2f,g). In addition, the serum antibody concentrations from two groups on days 1, 2, 3, and 7 were calculated using the standard dose‐response curve from Figure S2a (Supporting Information). Interestingly, these results further suggest that a single injection of mRNab‐LNPs could produce a more robust and sustained level of anti‐mCD19 antibodies in vivo compared to the conventional antibody treatment (Figure 2h). Altogether, these results demonstrate the advantages and translational potential of mRNab‐LNPs in antibody production and pharmacokinetics in vivo.

### 2.3 MRL/lpr Mice Benefited from CD19‐Targeted Therapeutics by Monoclonal Antibodies

To evaluate the efficacy of conventional CD19‐targeting monoclonal antibodies in autoimmunity diseases, we generated a mouse anti‐mouse CD19 IgG1 and investigated its in vivo effect using the spontaneous lupus‐prone murine models, MRL/lpr mice, for further investigation. The 12‐week‐old MRL/lpr mice were treated with isotype mouse IgG or anti‐mCD19 antibodies of 2.5mg kg^−1^ every three days for 4 weeks. MRL/mpj mice treated with isotype mouse IgG served as control (Figure S5a, Supporting Information). The percentages of the circulating CD45+CD19+ B cells from three groups were measured by flow cytometry after treatment, which confirmed that the anti‐mCD19 antibodies successfully depleted the circulating B cells in peripheral blood (Figure S5b,c, Supporting Information). Surprisingly, the volumes of the spleen were significantly reduced in lupus mice after the treatment of the anti‐mCD19 antibodies (Figure S5d,e, Supporting Information). We further performed immunohistochemistry (IHC) staining of spleens, lymph nodes, and Peyer's patches to detect the expression levels of CD20+ B cells. Similarly, the expression of CD20+ B cells were significantly decreased after the treatment of anti‐mCD19 antibodies (Figure S5f,g, Supporting Information). Notably, the co‐staining of CD19 with CD138 in the spleens of three groups showed that anti‐mCD19 antibodies were able to target and decrease the CD19+CD138+ plasma cells in lupus mice (Figure S5h,i, Supporting Information). These results indicate that the monoclonal anti‐CD19 antibodies are effectively deplete not only circulating B cells but also tissue‐resident plasma cells.

To further investigate the protective effects of the monoclonal anti‐CD19 antibodies on lupus mice after the depletion of CD19+ B cells and plasma cells, we evaluated the histological and pathological changes of skin and kidneys in three groups. Spontaneous skin lesions, represented by epidermal hyperplasia and the immune cell infiltration, were observed in MRL/lpr mice treated with the isotype IgG. Interestingly, treatment with anti‐CD19 antibodies significantly reduced these spontaneous skin lesions in MRL/lpr mice (Figure S6a, Supporting Information). Similarly, the histopathological changes, including glomerulonephritis and renal fibrosis, in kidneys were also significantly decreased in MRL/lpr mice treated with anti‐CD19 antibodies compared to those treated with isotype IgG (Figure S6b,c, Supporting Information). The IHC staining further showed that the immune cell infiltration was significantly reduced after the treatment with anti‐CD19 antibodies (Figure S6d,e, Supporting Information). In addition, the immunostaining of IgG and C3 showed that the deposition of the immune complex was also significantly reduced (Figure S6f,g, Supporting Information). Furthermore, treatment with anti‐CD19 antibodies significantly decreased the serum levels of anti‐dsDNA autoantibodies, 24‐h urinal albumin, blood urea nitrogen (BUN), and creatinine in MRL/lpr mice (Figure S6h–j, Supporting Information). Collectively, these results demonstrated that the monoclonal anti‐mCD19 antibodies have a strong depletion effect on B and plasma cells, and protect lupus mice from skin and kidney damage in vivo.

### 2.4 mRNab‐LNPs Successfully Depleted CD19+ B Cells and CD19+CD138+ Plasma Cells in MRL/lpr mice

**Figure 3:**
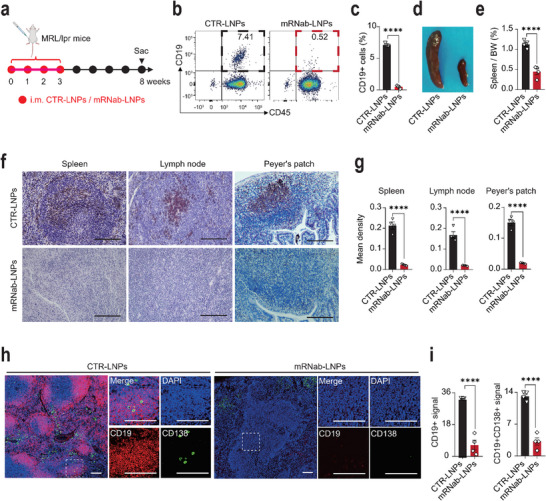
mRNab‐LNPs effectively depleted CD19+ B cells and plasma cells in MRL/lpr lupus mice. a) Schematic diagram of the experimental design for investigating the effects of mRNab‐LNPs on MRL/lpr lupus mice. The 12‐week‐old MRL/lpr female lupus mice were intramuscularly injected with empty LNPs or mRNab‐LNPs (1 mg kg^−1^) every week for 4 weeks consecutively. Blood and tissue samples were collected 5 weeks after the completion of the four administrations. b) Flow cytometry data showing the percentages of circulating CD45+CD19+ B cells in the MRL/lpr mice treated with empty LNPs or mRNab‐LNPs, respectively (*n* = 4 each). c) Quantitative results of the percentages of circulating CD45+CD19+ B cells from (b). d) Representative images of spleens from MRL/lpr mice treated with empty LNPs or mRNab‐LNPs, respectively (*n* = 4 each). e) The spleen to body weight (BW) ratios from two groups (*n* = 4 each). f) Representative immunohistochemistry images showing the expression of CD20 in the spleens, lymph nodes and Peyer's patches from MRL/lpr mice treated with empty LNPs or mRNab‐LNPs, respectively (*n* = 4 each). g) Quantitative results of CD20 expression in the spleens, lymph nodes and Peyer's patches from (f). h) Representative immunofluorescence images showing the expression of CD19 and CD138 in the spleens of the MRL/lpr mice treated with empty LNPs or mRNab‐LNPs, respectively (*n* = 4 each). i) Quantitative results of CD19 and CD138 expression in the germinal centers of spleens from (h). Data are mean ± standard error of the mean. Scale bars = 100 µm. ****p < 0.0001.

Next, we examined the therapeutic effect of mRNab‐LNPs on the spontaneous lupus MRL/lpr mice in vivo. MRL/lpr mice were injected intramuscularly of 1mg kg^−1^ empty LNPs (CTR‐LNPs) or mRNA‐LNPs once a week for 4 weeks. The B cell and plasma cell depleting effects of mRNab‐LNPs were then investigated 5 weeks after the final injection (**Figure** **3**a). The serum concentrations of mRNA‐encoding anti‐mCD19 antibodies in MRL/lpr mice at the endpoint were determined using a standard dose‐response curve, which demonstrated a significant increase in serum antibody levels in MRL/lpr mice treated with mRNA‐LNPs compared to the empty‐LNPs group (Figure S7, Supporting Information). The flow cytometry results showed that the population of circulating CD45+CD19+ and CD45+B220+ B cells was completely depleted in MRL/lpr mice treated with mRNab‐LNPs compared to that of MRL/lpr mice treated with CTR‐LNPs (Figure 3b,c; Figure S8, Supporting Information). In contrast, the percentages of CD3+ T cells and Ly6C+ monocytes remained comparable between the two groups (Figure S8, Supporting Information). Meanwhile, the spleen/body weight (BW) ratios were significantly reduced in mice treated with mRNab‐LNPs (Figure 3d,e). The IHC staining of another B cell marker protein CD20 indicated that B cells were almost completely depleted by mRNab‐LNPs in spleens, lymph nodes, and Peyer's patches of MRL/lpr mice (Figure 3f,g). Correspondingly, the immunofluorescence results of CD19 and CD138 showed that both CD19+ B cells and CD19+CD138+ plasma cells in the germinal centers were completely depleted in the spleens (Figure 3h,i). Altogether, these results suggest that mRNab‐LNPs could effectively and stably deplete B cells and plasma cells by producing anti‐mCD19 antibodies in MRL/lpr mice in vivo.

### 2.5 mRNab‐LNPs Protected MRL/lpr Mice from Skin and Renal Damages

**Figure 4:**
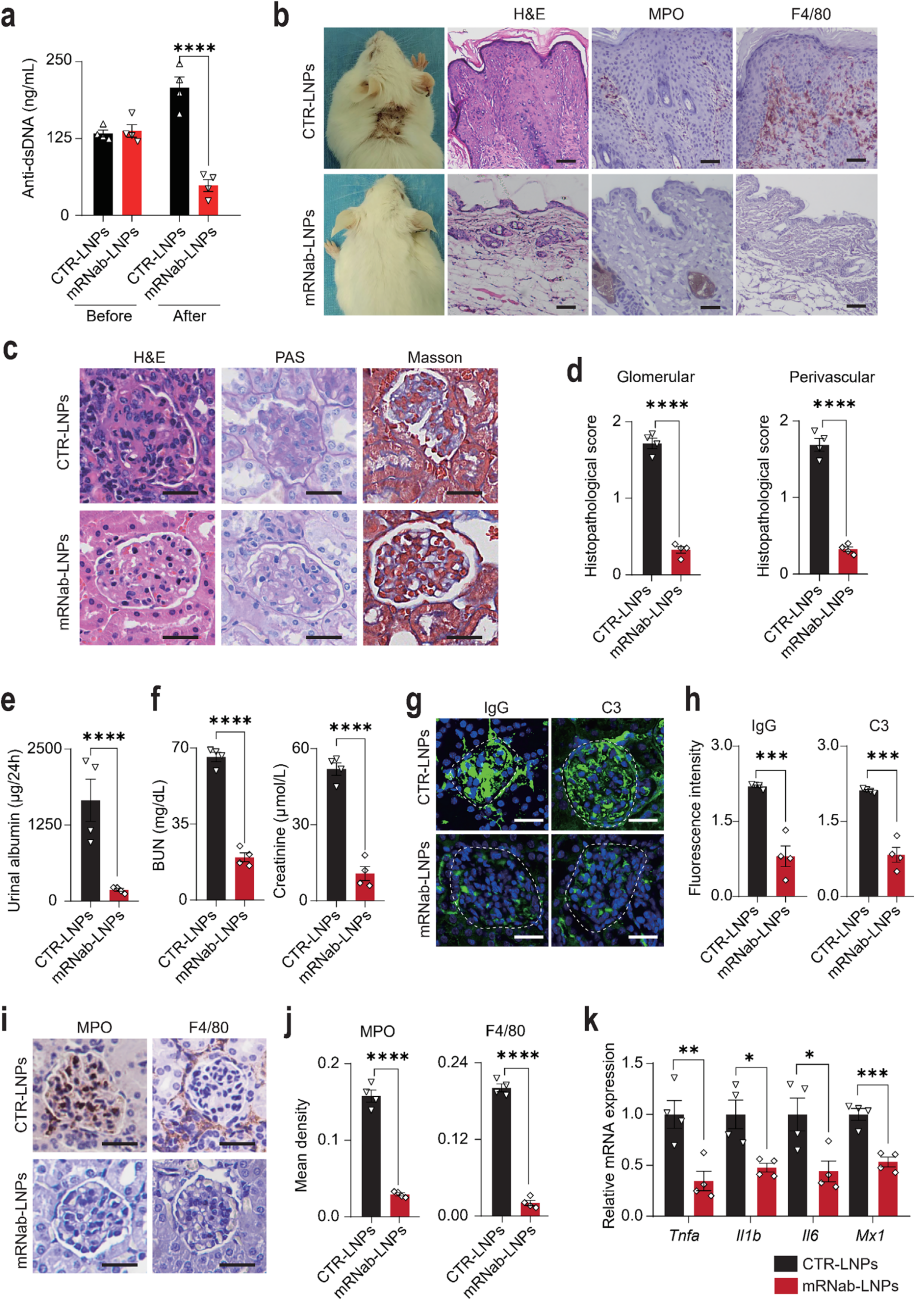
Depletion of B cells and plasma cells by mRNab‐LNPs protected MRL/lpr mice from skin damages and lupus nephritis. a) The levels of serum anti‐dsDNA antibody in MRL/lpr mice before and after the treatment of empty LNPs or mRNab‐LNPs (*n* = 4 each). b) Representative clinical, H&E staining, and immunohistochemistry images of the skin tissues from the MRL/lpr mice treated with empty LNPs or mRNab‐LNPs (1 mg kg^−1^), (*n* = 4 each). Scale bars = 50 µm. c) Representative H&E, PAS, and Masson staining images of the renal tissues from MRL/lpr mice treated with empty LNPs or mRNab‐LNPs (*n* = 4 each). Scale bars = 25 µm. d) Histopathological scores of glomerular and perivascular areas of the renal tissues from (c). e) The levels of 24‐h urinal albumin in MRL/lpr mice treated with empty LNPs or mRNab‐LNPs (*n* = 4 each). Scale bars = 25 µm. f) The levels of serum BUN and creatinine in MRL/lpr mice treated with empty LNPs or mRNab‐LNPs (*n* = 4 each). g) Representative immunofluorescence images of IgG and C3 in the renal tissues from the MRL/lpr mice treated with empty LNPs and mRNA‐LNPs, respectively (*n* = 4 each). Scale bars = 25 µm. h) Quantitative result of the expression levels of IgG and C3 from (g). i) Representative immunohistochemistry images of MPO and F4/80 in the renal tissues from MRL/lpr mice treated with empty LNPs or mRNA‐LNPs (*n* = 4 each). Scale bars = 25 µm. j) Quantitative result of the expression levels of MPO and F4/80 from (i). k) The mRNA expression levels of Tnfa, Il1b, Il6, and Mx1 in the renal tissues from the MRL/lpr mice treated with empty LNPs or mRNA‐LNPs (*n* = 4 each). Data are mean ± standard error of the mean. *p < 0.05, **p < 0.01, ****p < 0.001, **** *p* < 0.0001.

Autoantibodies secreted by autoreactive B cells and plasma cells, including anti‐dsDNA autoantibodies, play an important role in the autoimmunity of SLE patients.^[^ ^2b^ ^]^ We first examined the effect of mRNab‐LNPs on the depletion of anti‐dsDNA autoantibodies in animals. The levels of serum anti‐dsDNA autoantibodies in MRL/lpr mice were significantly increased before treatment, indicating that all MRL/lpr mice were in an active disease state (**Figure** **4**a). Following the treatment with mRNab‐LNPs, the serum levels of these autoantibodies were dramatically reduced in MRL/lpr mice (Figure 4a), which could be attributed to the depletion of B cells and plasma cells by mRNab‐LNPs. Spontaneous skin lesions and renal damages are the characterized features of lupus patients, which reflect the severity of autoimmunity in these patients.^[^ ^2a^ ^]^ Surprisingly, spontaneous skin lesions, characterized by epidermal hyperplasia and immune cell infiltration, were significantly reduced in MRL/lpr mice treated with mRNab‐LNPs compared to those treated with empty LNPs (Figure 4b). Western blotting analysis revealed a significant decrease in the expression levels of inflammatory cytokines, including TNF‐α, IL1β, IL6, and MX1, in the skin tissues of MRL/lpr mice treated with mRNab‐LNPs (Figure S9a,b, Supporting Information). Renal histopathological changes, including glomerulonephritis and fibrosis, were also significantly reduced in mice treated with mRNab‐LNPs (Figure 4c,d). In addition, mice treated with mRNab‐LNPs, but not CTR‐LNPs, showed a significant improvement in kidney functions, with lower levels of urinal albumin, serum BUN, and creatinine (Figure 4e,f). In addition, mRNab‐LNPs significantly attenuated the glomerular deposition of IgG and C3 in MRL/lpr mice (Figure 4g,h). These results suggest that depletion of B cells and plasma cells by mRNab‐LNPs could effectively decreased the immune complex deposition in glomeruli of lupus mice. Previous studies have shown that the immune complex deposition drives the infiltration and activation of innate immune cells in the kidney.^[^ ^20^ ^]^ We found that mRNab‐LNPs were effective in reducing the infiltration of MPO+ neutrophils, F4/80+ macrophages, and CD4+ T cells (Figure 4i,j; Figure S9c,d, Supporting Information), as well as the expression of pro‐inflammatory cytokines (Figure 4k; Figure S9e,f, Supporting Information), which may be due to the reduction of the immune complex deposition by mRNab‐LNPs in these mice. Taken together, these results suggest that mRNab‐LNPs could efficiently deplete the autoreactive B cells and plasma cells, reduce the autoantibodies, and attenuate the skin and renal damage in vivo.

### 2.6 mRNab‐LNPs were Effective Against Collagen‐Induced Arthritis in Mice

**Figure 5:**
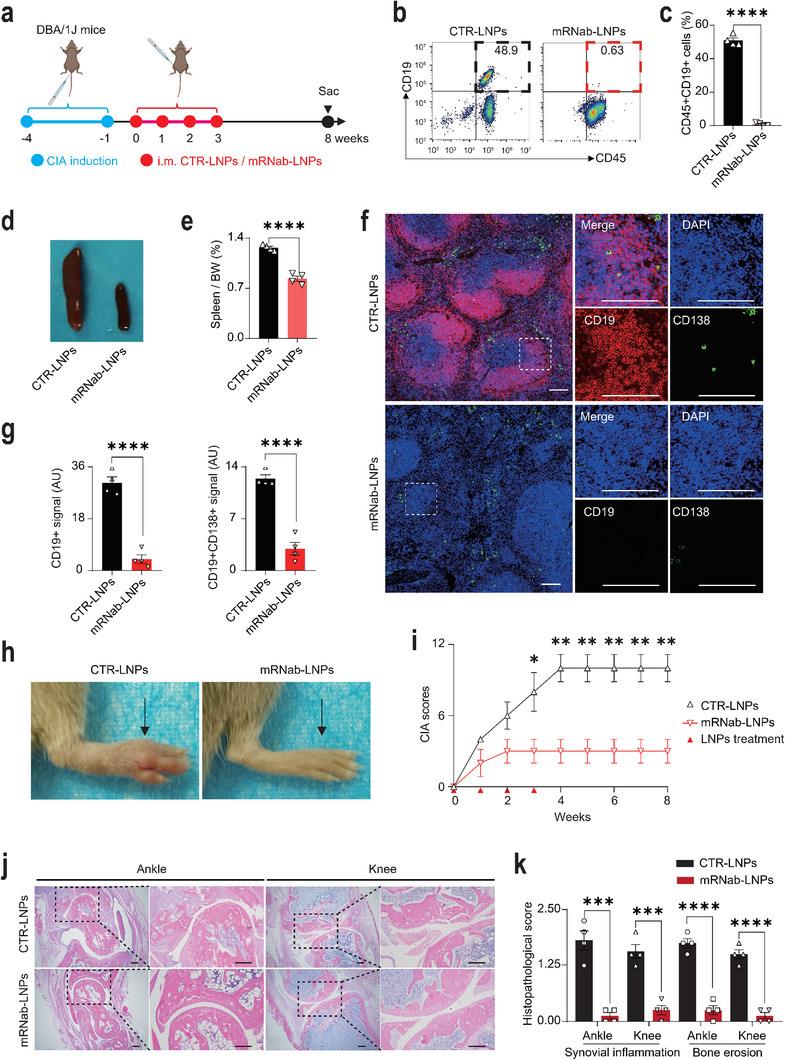
mRNab‐LNPs showed therapeutic benefits in collagen‐induced arthritis (CIA) mice. a) Schematic diagram of the experimental design for investigating the effects of mRNab‐LNPs on CIA mice. The DBA/1J mice were immunized with collagen II twice at 8‐ and 11‐week‐age. After one week, these immunized mice were intramuscularly injected with empty LNPs or mRNA‐LNPs (1 mg kg^−1^) every week for 4 weeks consecutively. Blood and tissue samples were collected 5 weeks after the fourth administration of LNPs. b) Flow cytometry data showing the percentages of circulating CD45+CD19+ B cells in CIA mice treated with empty LNPs or mRNA‐LNPs (*n* = 4 each). c) Quantitative results of the percentages of CD45+CD19+ B cell percentages from (b). d) Representative images of the spleens from CIA mice treated with empty LNPs or mRNA‐LNPs (*n* = 4 each). e) The spleen to body weight ratios from two groups (*n* = 4 each). f) Representative immunofluorescence images showing the expression of CD19 and CD138 in the germinal centers of spleens from CIA mice treated with empty LNPs or mRNA‐LNPs (*n* = 4 each). g) Quantitative results of CD19 and CD138 expression in the spleens from (f). h) Representative clinical images of the paws and knees in CIA mice treated with empty LNPs or mRNA‐LNPs (*n* = 4 each). i) Clinical scores of arthritis from CIA mice at the first administration of LNPs (week 0) and on week 1, 2, 3, 4, 5, 6, 7, and 8 after the first administration of LNPs (*n* = 4 each). j) Representative H&E images of the paws and knees from CIA mice treated with empty LNPs or mRNA‐LNPs (*n* = 4 each). k) Quantitative results of (j). Data are mean ± standard error of the mean. Scale bars = 100 µm. *p < 0.05, **p < 0.01, ***p < 0.001, ****p < 0.0001.

To further explore the therapeutic potential of mRNab‐LNPs in other autoimmune diseases, we examined the effect of mRNab‐LNPs in a collagen‐induced arthritis (CIA) mouse model, an autoimmune murine model of human RA (**Figure** **5**a). Similarly, serum levels of mRNA‐encoding anti‐mCD19 antibodies in CIA mice were measured using the indirect staining method. The results showed a significant increase in serum antibody levels in CIA mice treated with mRNab‐LNPs compared to the empty‐LNP group (Figure S10, Supporting Information). As expected, treatment with mRNab‐LNPs almost completely depleted the circulating CD19+ B cells in CIA mice (Figure 5b,c). The spleen/BW ratios were also reduced in CIA mice treated with mRNab‐LNPs (Figure 5d,e). Immunostaining results of CD19 and CD138 showed that both CD19+ B cells and CD19+CD138+ plasma cells in the germinal centers were completely depleted in the spleens of CIA mice treated with mRNab‐LNPs (Figure 5f,g). As expected, the CIA scores that were determined by the erythema and swelling of hind paws gradually increased in CIA mice. Notably, treatment with mRNab‐LNPs successfully attenuated the CIA scores by week 8 (Figure 5h,i). Histological results showed that treatment with mRNab‐LNPs significantly reduced the bone erosion in the ankle and knee joints of CIA mice (Figure 5j,k). In addition, there was a reduced infiltration of F4/80+ macrophage and CD4+ T cell in the joint tissues of treatment group (Figure S11, Supporting Information). These findings suggest that mRNab‐LNPs effectively suppress immune cell infiltration and inflammation in the affected joints.

To further evaluate the efficacy of mRNab‐LNPs, we treated RA mice with mRNab‐LNPs starting at 2 weeks, when the mice had developed well‐established arthritis. After four weekly injections, the mRNab‐LNPs treatment group demonstrated improved clinical scores, while the control group showed progression to more severe arthritis (Figure S12a,b, Supporting Information). Compared to mRNab‐LNPs, anti‐mCD19 antibody requires more frequent and higher number of injections to achieve a comparable therapeutic effect (Figure S13a,b, Supporting Information). Taken together, these results demonstrate the therapeutic potential of mRNab‐LNPs in various autoimmune diseases by depletion of B cells and plasma cells.

## 3 Discussion

SLE and RA are common autoimmune diseases in the clinic and can affect multiple organs as the disease progresses.^[^ ^2^, ^21^ ^]^ The immunological manifestations of the diseases are featured by the activation of autoreactive B cells and plasma cells, leading to the overproduction of autoantibodies.^[^ ^20^ ^]^ Recently, the field of immunotherapy is increasingly shifting from chronic immunosuppressive strategies toward approaches that aim to reset the immune system to a normal, tolerant state, thereby avoiding the long‐term deleterious effects associated with sustained immunosuppression.^[^ ^3^, ^22^ ^]^ However, there are still patients with refractory and relapsing disease.^[^ ^1^, ^23^ ^]^ Conventional monoclonal antibodies, which are composed of two full‐length heavy (H) and light (L) chains linked by charge‐charge interactions and disulfide bonds,^[^ ^16^ ^]^ have been rapidly developed for medical therapy, with promising results in autoimmune diseases, tumors, and infectious diseases.^[^ ^16^ ^]^ Although the latest guidelines have recommended antibodies targeting B cells, such as belimumab and rituximab, as add‐on therapies for the refractory and relapsing SLE and RA patients,^[^ ^24^ ^]^ results have not always been satisfactory. While current monoclonal antibodies for autoimmune diseases can deplete B cells, their efficacy varies widely and often fails to adequately target plasma cells.^[^ ^25^ ^]^ For example, obexelimab, a noncytolytic anti‐CD19 monoclonal antibody, failed to meet primary endpoints in a Phase II trial.^[^ ^4a^ ^]^ On the other hand, inebilizumab, an afucosylated anti‐CD19 antibody with enhanced antibody‐dependent cell‐mediated cytotoxicity (ADCC), required higher doses and repeated treatments to effectively deplete B cells and reduce autoantibodies in mice.^[^ ^26^ ^]^ In contrast, CD19‐targeted antibody‐drug conjugates, like mAb‐triptolide, showed improved cytotoxicity and better disease control in SLE models, underscoring the potential of CD19‐targeted therapies for treating autoimmune diseases.^[^ ^27^ ^]^ Recently, CAR‐T cell therapy has achieved ground‐breaking results in the treatment of autoimmune diseases, including SLE, idiopathic inflammatory myositis, and systemic sclerosis.^[^ ^11b^ ^]^ Treatment with CD19 CAR‐T cells has been shown to induce complete remission in SLE patients in several studies.^[^ ^8^, ^11^, ^25^ ^]^ Notably, a study revealed that protective IgG responses to standard vaccines—such as those for tetanus, measles, mumps, rubella, varicella–zoster virus, and Epstein–Barr virus—remained stable following CD19 CAR T‐cell therapy.^[^ ^11b^ ^]^ Furthermore, vaccinations administered after CD19 CAR T‐cell therapy and subsequent B‐cell reconstitution—including those for SARS‐CoV‐2, pneumococcus, and tetanus—elicited robust increases in antigen‐specific IgG levels.^[^ ^11b^ ^]^ Collectively, these studies have shown that CD19+ plasma cells, rather than CD19‐negative plasma cells, play a pivotal role in the pathogenesis of SLE. However, although CD19 CAR‐T cell therapy could adequately deplete disease‐associated antibody‐secreting B cells and short‐term plasma cells, the price and potential risk of T cell lymphoma limit its application in this area.

Recently, mRNA therapy emerges as a promising approach for protein and antibody production in vivo,^[^ ^28^ ^]^ which is capable of cell‐free batch production, rapid expression, repeated dosing, and well‐tolerated clinical safety concerns.^[^ ^15^ ^]^ Naked mRNAs are unstable and prone to degradation by ribonucleases and self‐hydrolysis, so encapsulating them in lipid nanoparticles (LNPs) has become a common strategy to protect mRNAs and enhance their intracellular delivery efficiency.^[^ ^29^ ^]^ To further improve the safety and tolerability of mRNA‐LNPs, various manufacturing strategies have been developed to reduce the activation of the innate immune response. For example, modifications to mRNAs—such as 5′ capping, nucleoside modification (e.g., uridine substitution with N1‐methyl‐pseudouridine), poly(A) tail modification, and HPLC purification—have been shown to effectively reduce innate immune activation.^[^ ^30^ ^]^ In addition, reducing the toxicity of lipid components, such as the development of ionizable lipids, is another important strategy for improving mRNA‐LNP tolerability.^[^ ^30^, ^31^, ^32^ ^]^ In clinical practice, pretreatment with immunosuppressants has also been used to minimize inflammatory responses.^[^ ^33^ ^]^ Beyond these approaches, emerging strategies utilizing tolerogenic nanoparticles offer the potential to restore immune tolerance without the need for immunosuppressive agents, further underscoring the promise of immune reset therapies.^[^ ^34^ ^]^ The first generation of mRNA‐LNP platforms has been successfully developed for COVID‐19 vaccines in humans, demonstrating overall safety with only mild local and systemic reactogenicity.^[^ ^30^, ^35^ ^]^ Moreover, recent studies have investigated the safety and durability of mRNA‐LNPs in humans across various diseases. For instance, the first‐in‐human study of passive immunotherapy targeting Chikungunya virus infection demonstrated the safety of mRNA‐LNPs, with reported adverse events being mild to moderate and transient, primarily consisting of infusion‐related reactions.^[^ ^33^ ^]^ Furthermore, mRNA‐LNP technology has been shown to enable the efficient delivery of pathogen‐specific IgA antibodies to mucosal surfaces, where they localize in the intestine, reduce bacterial invasion of Peyer's patches, and provide protection against lung and intestinal bacterial challenges.^[^ ^28^ ^]^ In addition, the first study of autologous RNA chimeric antigen receptor T‐cell therapy in an autoimmune disease was conducted in patients with generalized myasthenia gravis, which showed no dose‐limiting toxicities, cytokine release syndrome, or neurotoxicity.^[^ ^14^ ^]^ Notably, CPTX2309, an in vivo CAR‐T cell therapy delivering mRNA encoding an anti‐CD19 chimeric antigen receptor, selectively reprograms CD8+ cytotoxic T cells and demonstrates profound B cell depletion and immune reset potential in preclinical models. In non‐human primates, CPTX2309 induces rapid and deep B cell depletion followed by repopulation with predominantly naïve B cells within three weeks, a phenomenon consistent with observations in autoimmune disease patients treated with ex vivo CD19 CAR‐T cells, where naïve B cell return correlates with sustained remission and functional immune reset.^[^ ^11^ ^]^ Overall, mRNA‐LNP platforms have demonstrated safety, efficacy, and durability in the treatment of both infectious and autoimmune diseases. Our preclinical studies also showed that LNP‐encapsulated **mRN**A‐encoding **a**nti**b**odies (mRNab‐LNPs) did not significantly increase serum proinflammatory cytokines in MRL/lpr mice or cause adverse effects on body weight, liver, kidney function, or cause pathological changes in key organs of C57BL/6 mice. However, further study is needed to evaluate the long‐term safety, durability, and efficacy of these platforms in both preclinical and clinical settings.

To achieve deep depletion of tissue‐resident B and plasma cells, we developed an optimized LNP formulation with enhanced enrichment in secondary lymphoid organs, including spleens and lymph nodes, compared to other LNPs like SM‐102 and MC3. Western blotting confirmed that mRNab‐LNPs could induce robust protein production in these lymphoid tissues, demonstrating efficient immune modulation. In addition, our LNP formulation also exhibited high transfection efficiency across various human and mouse cell lines, highlighting its broad applicability and versatility. These findings support the therapeutic potential of mRNab‐LNPs, combining high efficacy with a favorable safety profile.

Inspired by the broad and efficient application prospects of CD19‐targeted therapeutics in autoimmunity diseases, we developed mRNab‐LNPs targeting CD19 for the depletion of B cells and plasma cells and explored their efficacy in MRL/lpr lupus mice and CIA mice models. Interestingly, intramuscular injection of mRNab‐LNPs resulted in high and sustained production of anti‐CD19 antibodies in mice. In particular, mRNab‐LNPs significantly reduced the numbers of CD19+ circulating B cells and tissue‐resident plasma cells in lupus and CIA mice. Subsequently, mice treated with the mRNab‐LNPs exhibited significantly reduced histopathological scores and injuries in skin, kidney, and joint. While the MRL/lpr lupus model remains a valid and widely used system for studying lupus pathogenesis, it is important to acknowledge the limitations of the CIA mice models in fully recapitulating the complexity of rheumatoid arthritis. In human RA, the dysregulated synovial microenvironment, characterized by the escape of inflammatory synovial cells from immunologic control, plays a central role in driving chronic inflammation and joint destruction.^[^ ^1^, ^21^ ^]^ Consequently, effective therapeutic strategies for human RA will likely require a combination of immunotherapy to target autoreactive immune cells and potent anti‐inflammatory agents to address the persistent synovial inflammation.^[^ ^24b,c^ ^]^ This dual approach may be essential to achieve sustained remission and prevent disease progression.^[^ ^1b^ ^]^ In light of these considerations, our findings with mRNab‐LNPs highlight the potential of transient and targeted B cell depletion as a therapeutic strategy for RA. In conclusion, our results demonstrate the therapeutic and translational advantages of mRNab‐LNPs in the treatment of autoimmune diseases.

## 4 Experimental Section

### Mice

Animal experiments were approved by the Institutional Animal Care and Use Committees of Sun Yat‐Sen University (approval No. SYSU‐IACUC‐2024‐001819). The 8‐week‐old female C57BL/6J mice were purchased from the Guangdong Medical Laboratory Animal Center, the 12‐week‐old female MRL/lpr mice were purchased from Jiangsu Huachuang Xinnuo Medical Technology Co., LTD and the 8‐week‐old male DBA/1J mice were purchased from Gempharmatech Co., Ltd. All the mice were housed in a pathogen‐free environment under a 12:12 h light‐dark cycle with the optimal humidity of 60–80% and the temperature of 22 ± 1 °C. No more than five mice were housed in each cage. The total volume of 24‐h urine samples from MRL/lpr mice was collected and the concentration of urinary albumin from MRL/lpr mice was determined using Coomassie Brilliant Blue staining with albumin standards. Serum samples from C57BL/6J mice and MRL/lpr mice were collected for the measurement of BUN, CREA, ALT, and AST using a fully automated biochemical analyzer (Hitachi 3100)

### Induction and Evaluation of Collagen‐Induced Arthritis in DBA/1J Mice

The RA mouse model was generated according to previous reports.^[^ ^36^ ^]^ Briefly, 100 µg chicken type II collagen (Chondrex) was emulsified with an equal volume (50 µL) of complete Freund's adjuvant (Chondrex). The emulsion was administered subcutaneously to the tail of 8‐week‐old male DBA/1J mice. Three weeks later, the mice were injected subcutaneously with 100 µg chicken type II collagen emulsified with 50 µL incomplete Freund's adjuvant (Chondrex) in a tail region remote from the initial injection site. Clinical manifestations of arthritis were scored as previously described.^[^ ^36a^ ^]^ Briefly, joint damage was assessed and graded as follows: 0, no obvious erythema and swelling; 1, erythema and mild swelling limited to the tarsal or ankle joint; 2, erythema and mild swelling from the ankle to the tarsal joint; 3, erythema and moderate swelling from the ankle to the metatarsal joints; 4, erythema and severe swelling involving the ankle, foot, and fingers, or limb ankylosis. The sum of the scores from four paws was defined as the final score, resulting in a maximum score of 16 per mouse. Histological analysis was performed to assess synovitis, cartilage damage, and bone erosion using a four‐point scale, with 0 indicating normal tissue, 1 indicating mild arthritis, 2 indicating moderate arthritis, and 3 indicating severe arthritis, as previously reported.^[^ ^37^ ^]^

### Design and Generation of mRNAs

Using the pMRNAxp vector (System Biosciences), the last base of the T7 promoter was modified to A and the polyA tail was extended to 120 bp, followed by the addition of a BspQI restriction enzyme site. The heavy and light chain fragments were cloned into the pMRNAxp vector linearized with EcoRI and BamHI restriction enzymes (Thermo Fisher Scientific) using the ClonExpress Ultra One Step Cloning Kit (Vazyme). The plasmid was amplified in E.coli and extracted using the TIANprep Mini Plasmid Kit (TIANGEN). The plasmids were linearized using BspQI restriction enzyme (New England Biolabs) or PCR (TransStart FastPfu DNA Polymerase, TransGen Biotech), and used as templates for in vitro transcription using an in vitro transcription kit (Changchun Golden Transfer Science and Technology). The synthesized mRNAs were purified using purification beads (VAHTS RNA Clean Beads, Vazyme), and the concentration and purity of the mRNAs were measured using a UV spectrophotometer.

### Construction of LNP‐Encapsulated mRNAs

An ethanol dilution method was used for LNP‐encapsulated mRNAs s processing. Briefly, the LNPs were formulated with a mixture of an ionizable lipid, structural lipid (cholesterol), phospholipid (DSPC), and helper lipid (DMG‐PEG2000) at a molar ratio of 16:12:8:4 in an alcohol solution. Firefly luciferase‐encoding mRNAs and anti‐mouse CD19 antibody‐encoding mRNAs were dissolved in 25 mm sodium acetate buffer solution. The ethanol phase of LNPs and the aqueous phase of mRNA were then vortexed at a volume ratio of 1:3. The mixtures were dialyzed with ultrapure water at room temperature for over 2 h.

### Characterization of mRNab‐LNPs

The morphology of LNPs was visualized using a transmission electron microscopy (JEOL JEM‐1200EX). For biophysical characterization, LNPs were diluted 1:100 with deionized water and Dynamic light scattering (DLS) was measured on a Zetasizer Nano ZS. To assess stability, the hydrodynamic diameter and polydispersity index (PDI) of LNPs stored at 4 °C for 7 days were measured at 25 °C using Zetasizer Nano ZS (Malvern Instruments). The sample was then diluted 1:100 with deionized water in a folded capillary colorimetric dish and the zeta potentials were measured on a Zetasizer Nano ZS at an applied voltage of 150 V.

### In Vivo Administration of mRNab‐LNPs

The 12‐week‐old female MRL/lpr mice and the 12‐ and 14‐week‐old immunized male DBA/1J mice were intramuscularly injected with mRNab‐LNPs (1mg kg^−1^) or an equal volume of empty LNPs in the left lower limb once a week for 4 weeks consecutively.

### In Vivo and Ex Vivo Bioluminescence Images

The 8‐week‐old female C57BL/6 mice were intramuscularly injected with 2 ug Firefly luciferase‐encoding mRNA‐LNPs (Fluc mRNA‐LNPs) and the bioluminescence images were captured using an in vivo imaging system (IVIS) platform (Tanon ABL X5 Pro). Prior to the imaging, each mouse was intraperitoneally injected with 100 µl D‐luciferin (15 mg mL^−1^, PerkinElmer) and then placed on the imaging platform under anesthesia with isoflurane (RWD Life Science). Bioluminescence images were acquired at 6 h, 12 h, 1d, 2d, 3d, 4d, 5d, 6d, and 7d after the Fluc mRNA‐LNPs injection. For the tissue distribution, the ex vivo bioluminescence images of major organs, including the brain, heart, lung, liver, spleen, lymph nodes, kidney, and muscle tissues, were taken 12 h after the Fluc mRNA‐LNPs injection.

### Flow Cytometric Analysis

Whole blood samples from MRL/lpr mice and CIA mice were treated with erythrocyte lysis buffer for 10 min at room temperature and then centrifuged at 2000 rpm for 5 min. The sedimented cells were washed once with PBS and incubated with Percpcy5.5‐anti‐CD45, PE‐anti‐CD19 antibodies, AF700‐anti‐B220, FITC‐anti‐CD3e, and PE‐anti‐Ly6C antibodies for 15 min at room temperature in the indicated experiments. To assess the specificity of the produced antibody, splenocytes of C57BL/6 mice were initially treated with an Fc receptor (FcR) blocker at 4 °C for 5 min. After washing, the cells were incubated at 37 °C for 1 h with two groups of conditioned media obtained from HEK293T cells transfected with mRNA‐LNPs for 0 and 48 h. The cells were then stained with AF647 anti‐mouse IgG, Percpcy5.5‐anti‐CD45, BV421‐anti‐B220, FITC‐anti‐CD3, PE‐anti‐Ly6c, and BV510‐anti‐CD56 antibodies for 15 min at room temperature. The cells were then analyzed using a flow cytometer (BD Biosciences) or CytoFLEX LX (Beckman).

A20 cells were incubated with conditioned media from mRNab‐LNPs‐transfected HEK293T cells or incubated with serums from anti‐mCD19‐antibodies‐treated and mRNab‐LNPs‐treated C57BL/6 mice for 1 h at room temperature. These anti‐mCD19 antibody‐labeled A20 cells were then washed once with PBS and stained with the second antibodies, AF647‐conjugated anti‐mouse IgG antibody for 15 min at room temperature. Untreated A20 cells were used as a blank control and A20 cells stained with a commercially available AF647‐conjugated anti‐mCD19 antibody were used as positive control. The stained A20 cells were analyzed using a flow cytometer (BD Biosciences) or CytoFLEX LX (Beckman). FlowJo software (Tree Star) was used for data analysis. Antibody information is provided in Table S1 (Supporting Information).

### Histological Analysis of Renal Tissues

Renal tissues from C57BL/6 mice and MRL/lpr mice were fixed in 4% paraformaldehyde and embedded in paraffin. For pathological evaluation, tissue sections were used for hematoxylin and eosin (H&E), periodic acid‐Schiff base (PAS), and Masson's trichrome staining according to the manufacturer's instructions. Pathological findings were graded on a scale of 0–3 as previously described.^[^ ^38^ ^]^ Briefly, histopathological findings of glomerular damage were graded on a scale of 0–3, with 0 indicating normal, 1 indicating mild cell proliferation and/or infiltration, 2 indicating moderate cell proliferation and/or infiltration, and 3 indicating severe cell proliferation and/or infiltration, membrane proliferation, and crescent formation and/or hyalinosis. Renal vascular lesions were graded on a scale of 0–3, with 0 indicating normal, 1 indicating mild perivascular cell infiltration, 2 indicating moderate destruction of the arterial wall, and 3 indicating severe thickening.

### Immunohistochemistry

Spleens, lymph nodes, Peyer's patches, skin, and kidney tissues from MRL/lpr mice were fixed in 4% paraformaldehyde and embedded in paraffin for immunohistochemical analysis. Joint tissues from CIA mice were fixed in 4% paraformaldehyde, decalcification in 0.5 m EDTA (pH 7.2) (G1105, Servicebio) for 4 weeks and embedded in paraffin for immunohistochemical analysis. Briefly, tissue sections were blocked with goat serum and incubated with a rabbit anti‐mouse CD20, rabbit anti‐mouse MPO, rabbit anti‐mouse F4/80, and rabbit anti‐mouse CD4 overnight at 4 °C. The sections were then stained with HRP‐conjugated anti‐rabbit IgG antibody, and visualized using the DAB kit (ZS). Quantification was performed using Image‐Pro Plus 6.0 (Media Cybernetics). The integrated optical density (IOD) was defined as the total number of positive materials in all locations within the region. The mean density in the selected area was calculated as IOD/area. Antibody information is provided in Table S1 (Supporting Information).

### Immunofluorescence

The spleens of MRL/lpr mice and CIA mice were fixed in 4% paraformaldehyde and embedded in paraffin for immunofluorescence analysis. Briefly, tissue sections were incubated with rabbit anti‐mouse CD138 overnight at 4 °C and were incubated with HRP‐conjugated goat anti‐rabbit IgG for 50 min the following day. The slides were then incubated for 10 min with iFluor 488 tyramide (Servicebio) for tyramide signal amplification (TSA). After antigen retrieval, the second primary antibodies, rabbit anti‐mouse CD19 antibodies, were added and CY3 goat anti‐rabbit IgG was used as the corresponding secondary antibody. DAPI was used for nuclear staining. Slides were visualized using a fluorescence microscope (Olympus IX73). For cell staining, A20 cells were processed and stained as described above (see Flow cytometric analysis). Nuclei were stained with DAPI and cells were imaged using fluorescence microscopy (Olympus IX73). Antibody information is listed in Table S1 (Supporting Information).

### De‐Glycosylation of Antibody

The protein samples were de‐glycosylated using the Peptide N‐Glycosidase F (PNGase F) De‐Glycosylation Kit (P2318S, Beyotime, China) following the manufacturer's protocol. Briefly, protein samples were denatured by adding the denaturation buffer provided in the kit and heating at 100 °C for 10 min to expose glycosylation sites. After cooling to room temperature, PNGase F enzyme and the reaction buffer were added to the sample, and the mixture was incubated at 37 °C for 1 h. The resulting de‐glycosylated proteins were analyzed via SDS‐PAGE to confirm molecular weight shifts indicative of successful glycan removal.

### Western Blot Analysis

Tissue and cell lysates were prepared using a radioimmunoprecipitation assay (RIPA) lysis buffer (CWBIO) supplemented with protease and phosphatase inhibitors (Beyotime). The lysates were centrifuged at 13 000 g for 15 min at 4 °C. The supernatants were then collected for the measurement of protein concentration using bicinchoninic acid (BCA) assay (ThermoFisher). Anti‐TNFα, anti‐IL1β, anti‐IL6, anti‐MX1, HRP‐conjugated anti‐mouse IgG antibodies, rabbit anti‐GAPDH antibodies, and HRP‐conjugated goat anti‐rabbit IgG antibodies were used for immunoblot analysis. Signals were observed using chemiluminescence ECL substrate (Merck Millipore). Details of the antibodies are given in Table S1 (Supporting Information).

### Enzyme‐Linked Immunosorbent Assay (ELISA)

The serum levels of anti‐double‐stranded DNA antibodies (anti‐dsDNA), TNF‐α, IL1β, and anti‐IL6 of MRL/lpr mice were measured using ELISA kits (Cusabio; Servicebio) following the manufacturer' protocols. Absorbance was determined at an optical density of 450 nm.

### Quantitative Real‐Time PCR

Total RNA was extracted from renal tissues using EZ‐press RNA Purification Kit (EZBioscience) according to the manufacturer's instructions. cDNA was synthesized using Color Reverse Transcript Kit (EZBioscience). RT‐qPCR assay was performed using SYBR Green Color qPCR Mix (EZBioscience) on an Azure Cielo real‐time PCR system. The comparative threshold cycle (Ct) value of each sample was calculated, and the relative mRNA expression was normalized to the Gapdh value. Gene‐specific primers are listed in Table S2 (Supporting Information).

### Cell Culture and Transfection

All cells were purchased from ATCC. Human embryonic kidney‐derived 293T (HEK293T) cells, Hela cells, and HuH‐7 cells were maintained in DMEM (Genom) supplemented with 10% fetal bovine serum (ExCell Bio) and 100 U mL^−1^ penicillin and 100 µg mL^−1^ of streptomycin (HyClone) in a humidified CO_2_ incubator. A20 cells were maintained in RPMI 1640 (GIBCO) supplemented with 10% fetal bovine serum and 100 U mL^−1^ penicillin and 100 µg mL^−1^ of streptomycin in a humidified CO_2_ incubator. Cells were grown in a six‐well plate and 400 ng of mRNab‐LNPs per well was added for transfection. HEK293T, Hela, HuH‐7, and A20 cells were harvested 24 h after the transfection for Western blotting of the heavy and light chains of anti‐mCD19 mouse IgG. Conditioned media of HEK293T cells were collected 6, 12, 24, and 48 h after transfection with mRNab‐LNPs.

### Statistical Analysis

All data are presented as mean ± SEM and analyzed using GraphPad Prism 8.0 (GraphPad Software). Student's *t*‐test was used to compare two groups, and one‐way analysis of variance (ANOVA) with Dunnett's multiple comparison test was used to compare more than two groups. A *p*‐value less than 0.05 was considered statistically significant.

## Conflict of Interest

The authors declare no conflict of interest.

## Author Contributions

Y.Z., J.C., J.W., and L.W. conceived the idea and designed the experiments. C.G. performed most of the in vitro, in vivo experiments, analyzed, generated data, with assistance from X.Y., S.L., Y.D., and L.W. L.Z. and Y.T. constructed the mRNab‐LNPs. C.G. and Y.Z. wrote the manuscript and all authors critically reviewed the manuscript. All authors agree to publish this manuscript.

## Supporting information



Supporting Information

## Data Availability

The data that support the findings of this study are available from the corresponding author upon reasonable request.
