# Supplementary material for: Therapeutic Potential of Lipid Nanoparticle‐Encapsulated CD19‐Targeting mRNAs in Lupus and Rheumatoid Arthritis
Source: Adv Sci (Weinh). 2025 Apr 7;12(21):2501628. doi: 10.1002/advs.202501628 (PMC12140323; doi:10.1002/advs.202501628)

Therapeutic Potential of Lipid Nanoparticle-Encapsulated CD19-Targeting mRNAs in Lupus and Rheumatoid Arthritis

*Chipeng Guo, Yingsen Tang, Ling Zeng, Xiaomei You, Siweier Luo, Yufei Du, Le Wang, Liangchun Wang, Jianchuan Wang*, Jinjin Chen*, Yiming Zhou**

C. Guo, X. You, L. Wang, Y. Zhou

Department of Dermatology

Sun Yat-sen Memorial Hospital

Sun Yat-sen University

Guangzhou, 510120, China

E-mail: [zhouym35@mail.sysu.edu.cn](mailto:zhouym35@mail.sysu.edu.cn)

Y. Tang, L. Zeng, S. Luo, Y. Du, L. Wang, J. Chen, Y. Zhou

Basic and Translational Medical Research Center

Sun Yat-sen Memorial Hospital

Sun Yat-sen University

Guangzhou, 510120, China

E-mail: [chenjj365@mail.sysu.edu.cn](mailto:chenjj365@mail.sysu.edu.cn)

J. Wang

School of Pharmaceutical Sciences

Sun Yat-sen University

Guangzhou, 510006, China

E-mails: [wangjch77@mail.sysu.edu.cn](mailto:wangjch77@mail.sysu.edu.cn)

**Table S1. List of antibodies used in the study.**

| **Antibody** | **Company** | **Clone** |  |
| --- | --- | --- | --- |
| PerCP-Cy5.5 Rat Anti-Mouse CD45 | BD | 550994 |  |
| ABflo® 647 Rabbit anti-Mouse CD19 | Abclonal | A24101 |  |
| FITC Hamster Anti-Mouse CD3e | BD | 553061 |  |
| Brilliant Violet 421 anti-Mouse CD45R/B220 | biolegend | 103239 |  |
| PE Rat Anti-Mouse Ly6C | BD | 560592 |  |
| BV510 Rat Anti-Mouse CD56 | BD | 748096 |  |
| Alexa Fluor® 700 anti-Mouse CD45R/B220 | biolegend | 103231 |  |
| Anti-CD19 | Servicebio | GB11061-1 |  |
| Anti-CD138 | Servicebio | GB115052 |  |
| HRP-conjugated Goat Anti-Rabbit IgG | Servicebio | GB23303 |  |
| CY3 Goat Anti-Rabbit IgG | Servicebio | GB21303 |  |
| DAPI | Abcam | ab188804 |  |
| Alexa Fluor® 647 anti-mouse IgG | Abcam | ab150115 |  |
| Anti-CD20 | Abcam | ab64088 |  |
| Anti-MPO | Abcam | ab208670 |  |
| Anti-F4/80 | Abcam | ab300421 |  |
| Anti-CD4 | Abcam | ab183685 |  |
| Anti-TNFα | Abcam | ab1793 | |
| Anti-IL1β | Abcam | ab283818 | |
| Anti-IL6 | Abclonal | A24522 | |
| Anti-MX1 | Abcam | ab222856 | |
| HRP-linked anti-mouse IgG H&L | CST | 7076S | |
| Anti-GAPDH | Abcam | ab8245 | |

**Table S2. List of primers used in real-time quantitative PCR.**

| Symbol | Forward Primer | Reverse Primer |
| --- | --- | --- |
| Gapdh | AGGTCGGTGTGAACGGATTTG | GGGGTCGTTGATGGCAACA |
| Il1b | GAAATGCCACCTTTTGACAGTG | TGGATGCTCTCATCAGGACAG |
| Il6 | CTGCAAGAGACTTCCATCCAG | AGTGGTATAGACAGGTCTGTTGG |
| Tnfa | CAGGCGGTGCCTATGTCTC | CGATCACCCCGAAGTTCAGTAG |
| Mx1 | GACCATAGGGGTCTTGACCAA | AGACTTGCTCTTTCTGAAAAGCC |

**Supplementary Figure 1. Analysis of antibody glycosylation.**

Western blotting analysis comparing the glycosylation profiles of conventional and mRNA-encoded anti-mCD19 antibodies, both with and without PNGase F enzymatic deglycosylation treatment.

**Supplementary Figure 2. Quantification of anti-mCD19 antibodies concentrations using a standard curve.**

**(a)** Generation of a standard curve for quantification of anti-mCD19 antibodies.

Mouse B cells were labeled with specific concentrations of anti-mCD19 antibodies (0.001, 0.01, 0.1, 1, 10, and 100 μg/ml) for 1 hour, and then incubated with a secondary antibody (AF647-conjugated anti-mouse IgG antibody) for the flow cytometry analysis. The y-axis is the percentage of labelled B cells, the x-axis is the logarithm of concentration of anti-mCD19 antibodies.

**(b)** The calculated concentrations of anti-mCD19 antibodies from supernatants of **Figure 1i** using the standard curve from **(a)** (n = 3 each).

Data are mean ± standard error of the mean.

*****p* < 0.0001.

**Supplementary Figure 3. Flow cytometry analysis of anti-mCD19 antibody specificity in mouse splenocytes.**

Mouse splenocytes were initially treated with an Fc receptor blocker at 4°C for 5 minutes. After washing, the cells were incubated at 37°C for 1 hour with two groups of conditioned media obtained from HEK293T cells transfected with mRNab-LNPs for 0 and 48 hours, respectively. Cells treated with conventional anti-mCD19 antibodies (100μg/ml) were used as a positive control.

**Supplementary Figure 4. The biocompatibility of LNPs *in vivo*.**

**(a)** The body weight from C57BL/6 mice one week after the treatment of PBS or LNPs (n = 4 each).

**(b)** Serological results of ALT, AST, BUN, and Cr from C57BL/6 mice one week after the treatment of PBS or LNPs (n = 4 each).

**(c)** Representative H&E images of vital organs from two groups (n = 4 each).

**(d)** The levels of serum TNFα, IL1β, and IL6 of MRL/lpr mice 72 hours after treated with empty LNPs (CTR-LNPs) or mRNab-LNPs (n = 4 each).

Scale bars = 50 μm.

**Supplementary Figure 5. The anti-mCD19 antibodies achieved a desired exhaustion of CD19+ B and plasma cells in MRL/lpr mice.**

**(a)** Schematic diagram of the experimental design for investigating the effects of anti-mCD19 antibodies on MRL/lpr mice. The 12-week-old MRL/lpr mice were intravenously injected with isotype mouse IgG or anti-mCD19 antibodies (2.5mg/kg) every three days for 4 weeks. The MRL/mpj mice injected with isotype mouse IgG served as control. Blood and tissue samples were collected at week 16 for further analysis.

**(b)** Flow cytometry data of percentages of circulating CD45+CD19+ B cells from three groups (n = 4 each).

**(c)** Quantitative results of the percentages of CD45+CD19+ B cells from **(b)**.

**(d)** Representative images of the spleens from three groups (n = 4 each).

**(e)** The spleen to body weight ratios from three groups (n = 4 each).

**(f)** Representative immunohistochemistry images showing the expression of CD20 in the spleens, lymph nodes and Peyer’s patches from three groups (n = 4 each).

**(g)** Quantitative results of the CD20 expression from **(f)**.

**(h)** Representative immunofluorescence images showing the expression of CD19 and CD138 in the spleens from three groups (n = 4 each).

**(i)** Quantitative results of the CD19 and CD138 expression from **(h)**.

Data are mean ± standard error of the mean.

Scale bars = 100 μm.

***p* < 0.01, *****p* < 0.0001.

**Supplementary Figure 6. Treatment with anti-mCD19 antibodies improved the skin and renal damages in lupus mice.**

**(a)** Representative H&E and immunohistochemistry images of the skin tissues from MRL/mpj mice treated with mouse isotype IgG, and MLR/lpr mice treated with mouse isotype IgG or anti-mCD19 antibodies (n = 4 each).

**(b)** Representative H&E, PAS, and Masson staining images of the renal tissues from three groups (n = 4 each).

**(c)** Histopathological scores of the renal tissues from **(b)**.

**(d)** Representative immunohistochemistry images of MPO and F4/80 in the renal tissues from three groups (n = 4 each).

**(e)** Quantitative result of MPO and F4/80 expression from **(d)**.

**(f)** Representative immunofluorescence images of IgG and C3 in the renal tissues from three groups (n = 4 each).

**(g)** Quantitative result of IgG and C3 expression from **(f)**.

**(h)** The levels of serum anti-dsDNA antibody from three groups (n = 4 each).

**(i)** The levels of 24-hour urinal albumin from three groups (n = 4 each).

**(j)** The levels of serum BUN and creatinine from three groups (n = 4 each).

Data are mean ± standard error of the mean.

Scale bars = 50 μm.

**p* < 0.05, ***p* < 0.01, ****p* < 0.001, *****p* < 0.0001.

**Supplementary Figure 7. Serum concentrations of anti-mCD19 antibodies** **at the study endpoint from MRL/lpr mice.**

Mouse B cells were incubated with serum collected at the endpoint from MRL/lpr mice treated with either empty LNPs or mRNab-LNPs. Serum concentrations of anti-mCD19 antibodies in the two groups were calculated using the standard curve shown in **Figure S2a** (n = 4 each).

Data are mean ± standard error of the mean.

*****p* < 0.0001.

**Supplementary Figure 8.** **Percentages of circulating immune cells at the study endpoint from MRL/lpr mice.**

**(a)** Gating strategy of flow cytometry.

**(b)** Flow cytometry data of percentages of circulating B220+ B cells, CD3+ T cells, and Ly6C+ monocytes from MRL/lpr mice treated with CTR-LNPs and mRNab-LNPs (n = 4 each).

**(c)** Quantitative results of the percentages of the cells from **(b)**.

Data are mean ± standard error of the mean.

*****p* < 0.0001.

**Supplementary Figure 9. Phenotypic validation of skin and renal tissues in MRL/lpr mice treated with empty LNPs and mRNab-LNPs.**

**(a)** The protein expression levels of TNF-α, IL1β, IL6, and MX1 in the skin tissues from the MRL/lpr mice treated with empty LNPs (CTR-LNPs) and mRNab-LNPs (n = 4 each).

**(b)** Quantitative result of **(a)**.

**(c)** Representative immunohistochemistry images of CD4 in the renal tissues from the MRL/lpr mice treated with empty LNPs (CTR-LNPs) and mRNab-LNPs (n = 4 each). Scale bars = 25 μm.

**(d)** Quantitative result of the expression levels of CD4 from **(c)**.

**(e)** The protein expression levels of TNF-α, IL1β, IL6, and MX1 in the renal tissues from the MRL/lpr mice treated with empty LNPs (CTR-LNPs) and mRNab-LNPs (n = 4 each).

**(f)** Quantitative result of **(e)**.

Data are mean ± standard error of the mean.

**p* < 0.05, ***p* < 0.01.

**Supplementary Figure 10. Serum concentrations of anti-mCD19 antibodies** **at the study endpoint from CIA mice.**

Mouse B cells were incubated with serum collected at the endpoint from CIA mice treated with either empty LNPs (CTR-LNPs) or mRNab-LNPs. Serum concentrations of anti-mCD19 antibodies in the two groups were calculated using the standard curve shown in **Figure S2a** (n = 4 each).

Data are mean ± standard error of the mean.

*****p* < 0.0001.

**Supplementary Figure 11. Representative immunohistochemistry images of F4/80 and CD4 in the ankle and knee joints from the CIA mice treated with empty LNPs (CTR-LNPs) or mRNA-LNPs.**

Scale bars = 25 μm.

**Supplementary Figure 12. Representative clinical images and scores of arthritis from established CIA mice treated with empty LNPs and mRNab-LNPs.**

**(a)** Treatment was initiated two weeks after the second immunization, a time when CIA mice had developed well-established arthritis. Representative clinical images of the paws and knees from CIA mice treated with either empty LNPs (CTR-LNPs) and mRNab-LNPs are shown. Images were captured at the time of the second immunization and at weeks 2 and 8 post-second immunization (n = 4 each).

**(b)** Clinical arthritis scores of CIA mice recorded at the time of the second immunization and weekly thereafter, from week 1 to week 8 post-second immunization (n = 4 each).

Data are mean ± standard error of the mean.

**p* < 0.05.

**Supplementary Figure 13. Representative clinical images and scores of arthritis from CIA mice treated with isotype mouse IgG and anti-mCD19 antibodies.**

**(a)** The CIA mice were intravenously injected with isotype mouse IgG or anti-mCD19 antibodies (2.5mg/kg) every three days for 4 weeks. Representative clinical images of the paws and knees in CIA mice treated with isotype IgG or synthetic anti-mCD19 antibodies (n = 4 each).

**(b)** Clinical scores of arthritis from CIA mice at the first administration of antibodies (week 0) and on week 1, 2, 3, 4, 5, 6, 7, and 8 after the first administration of antibodies (n = 4 each).

Data are mean ± standard error of the mean.

**p* < 0.05, ***p* < 0.01.

**Supplementary Figure 14.** **Full scans of western blots in Fig. 1f, 2d, S1, and S9.**


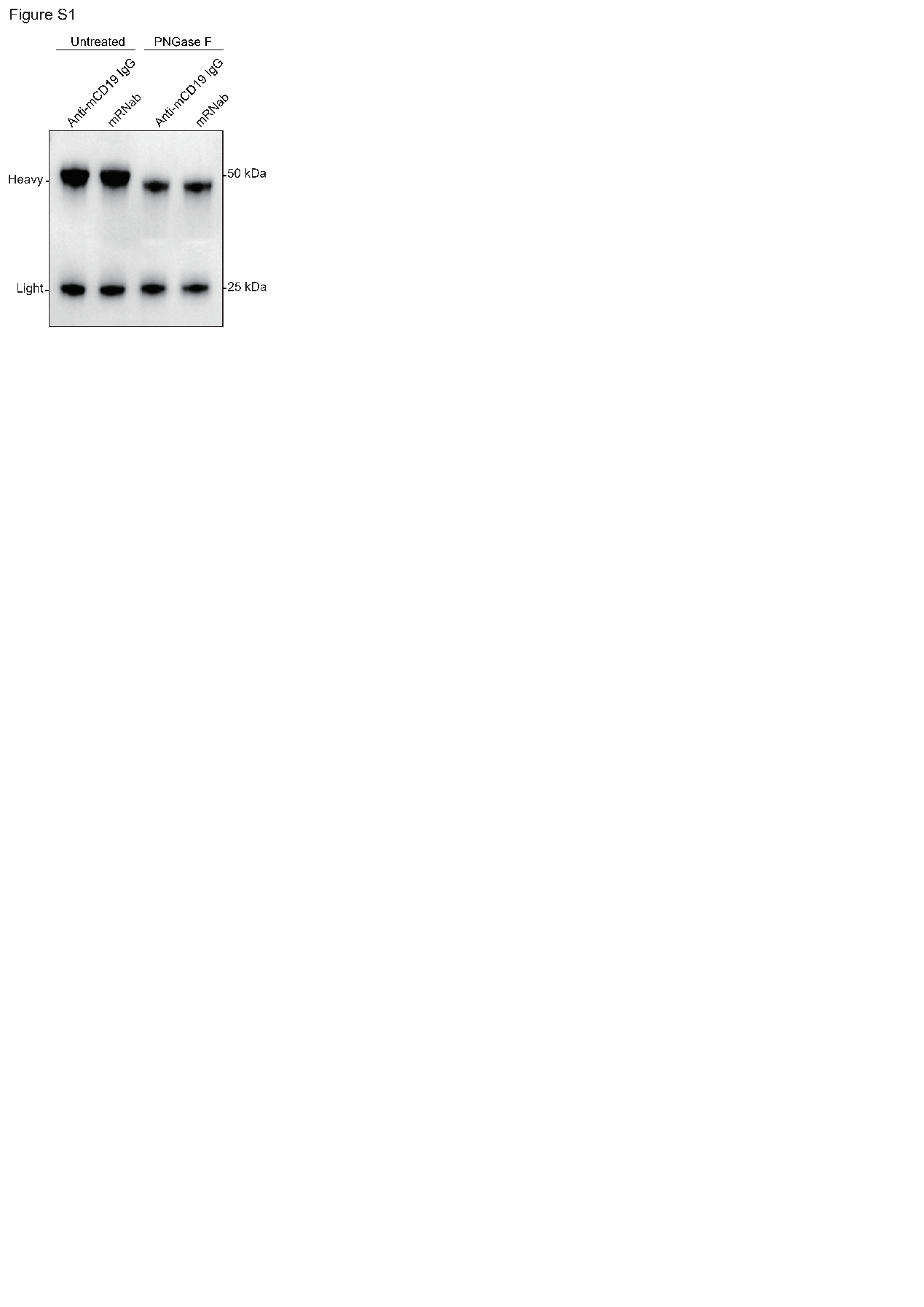

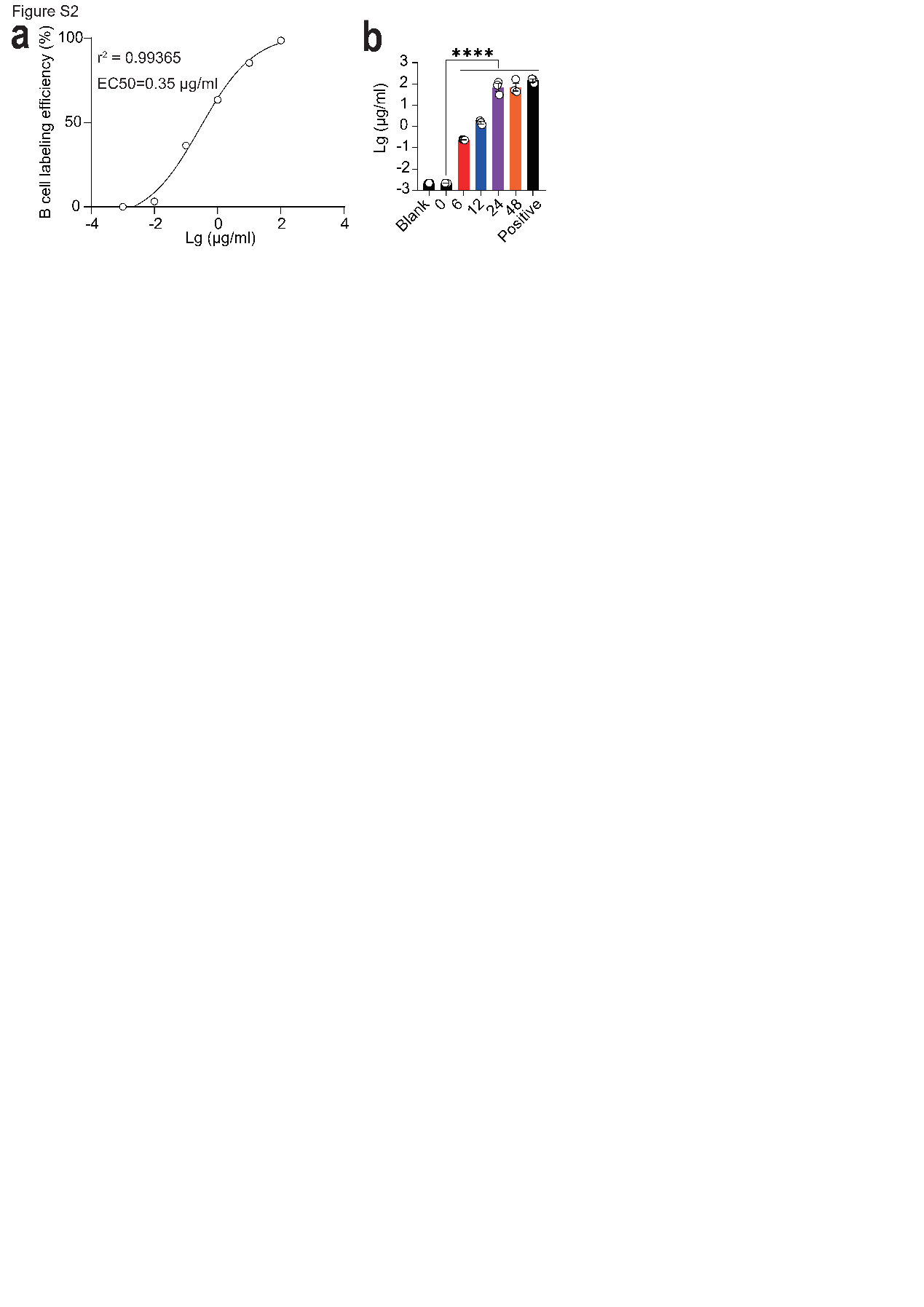

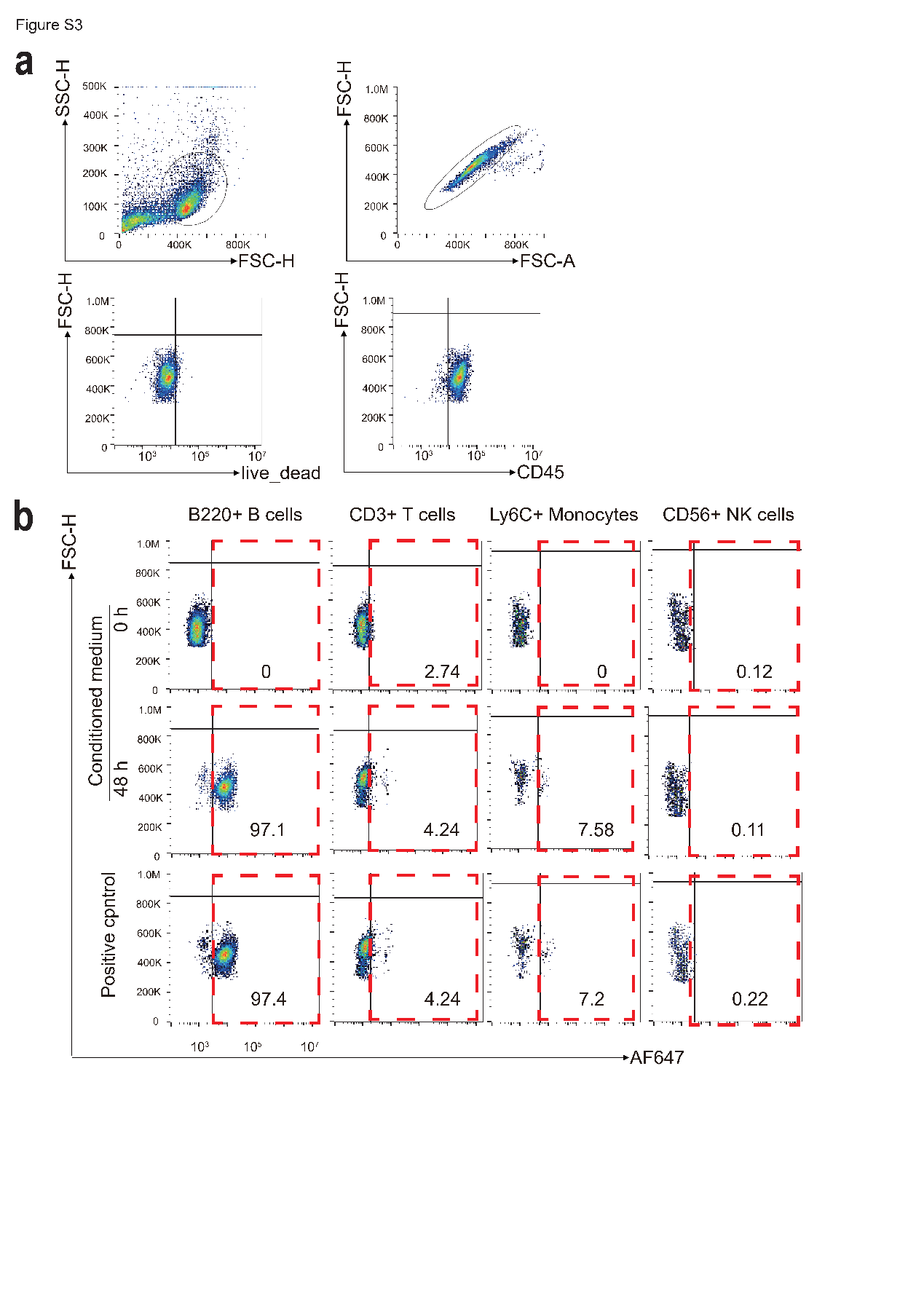

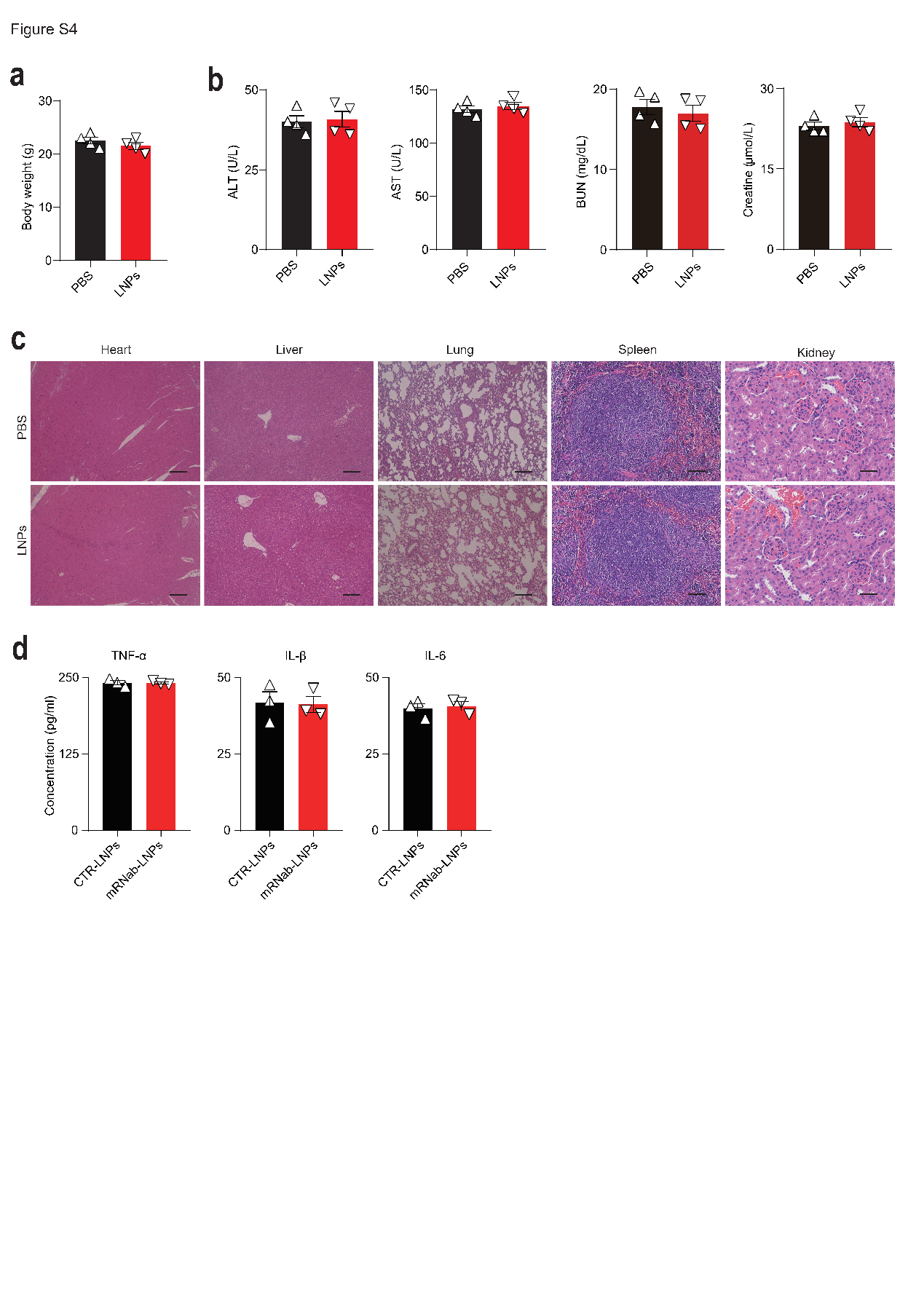

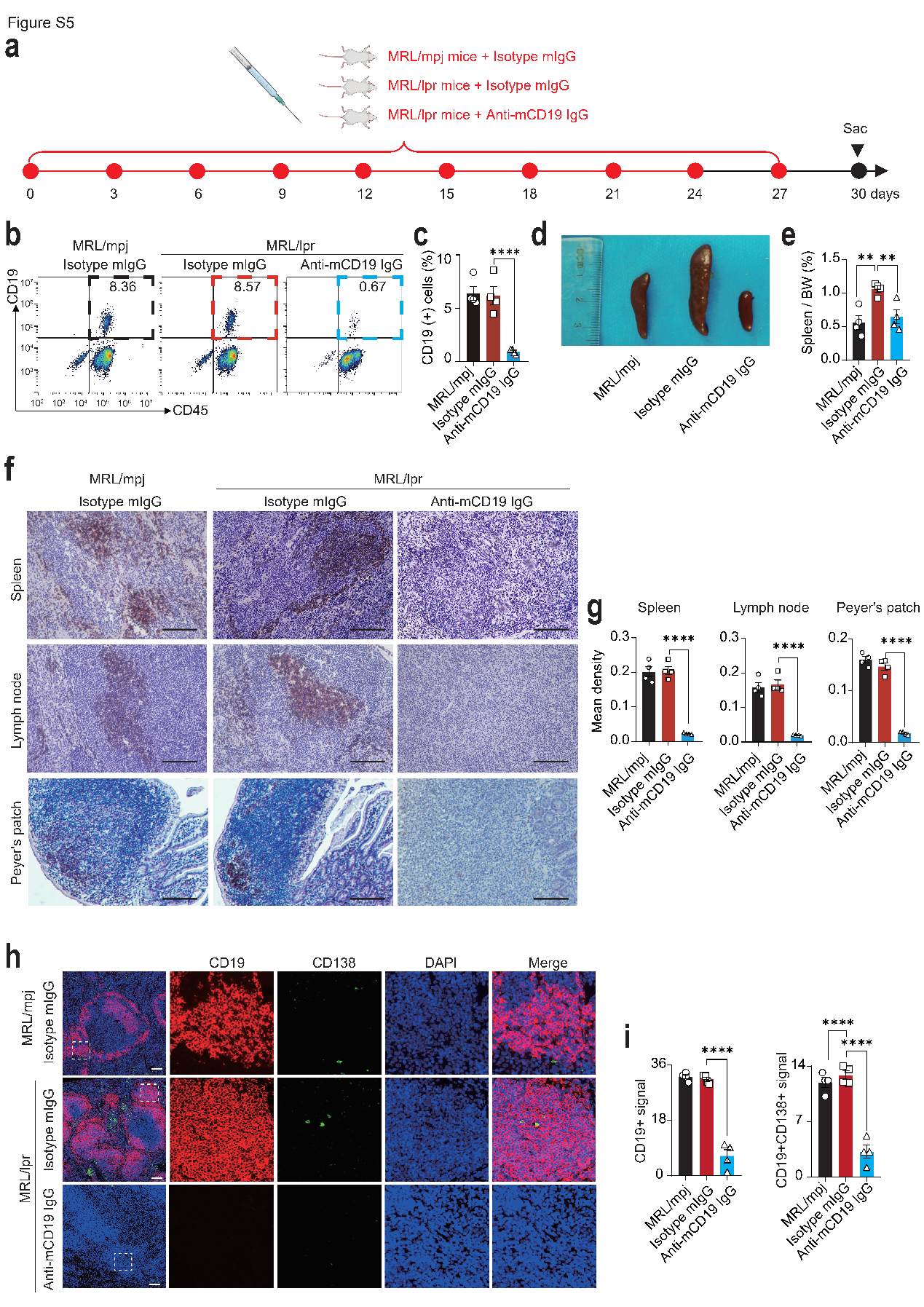

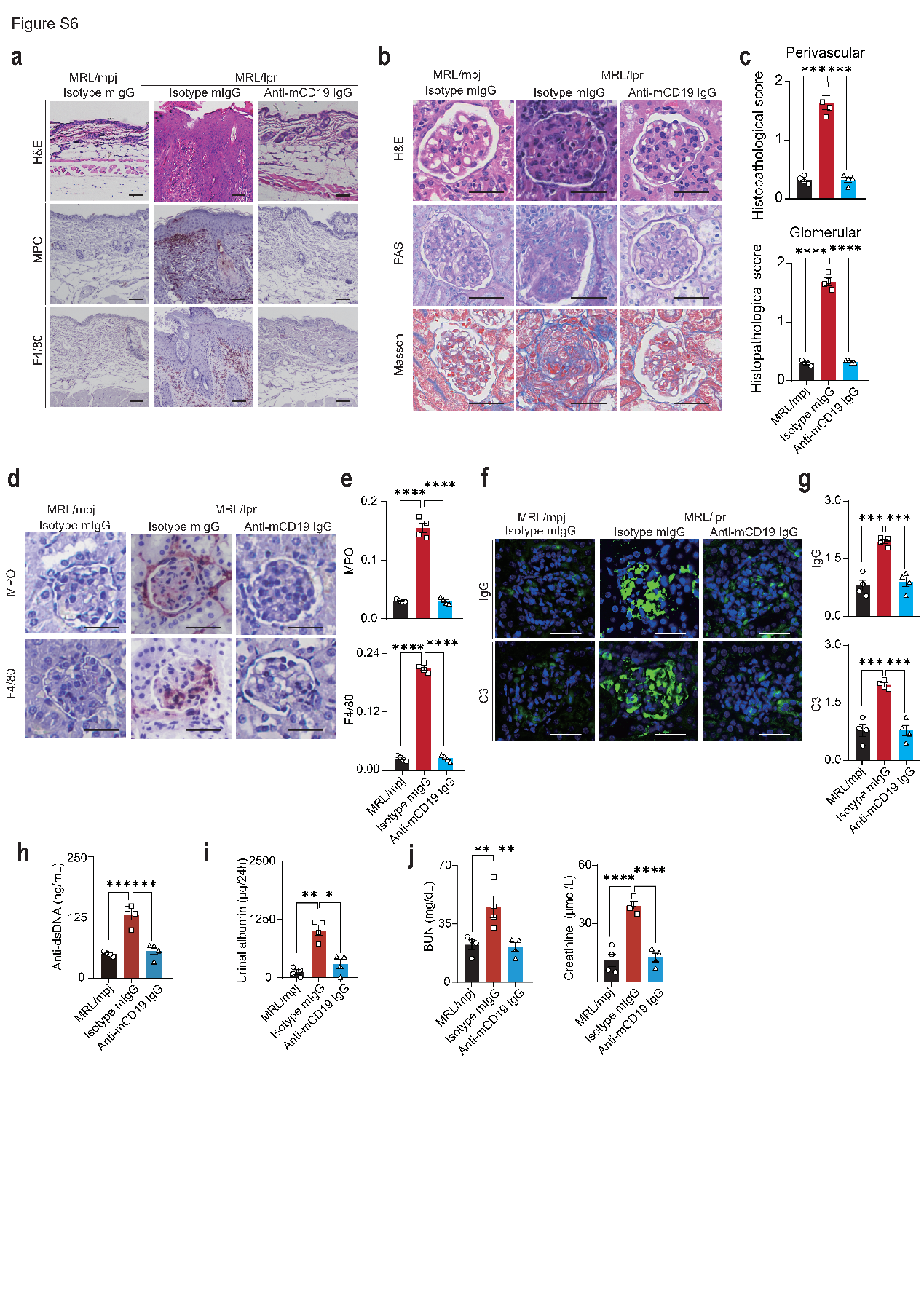

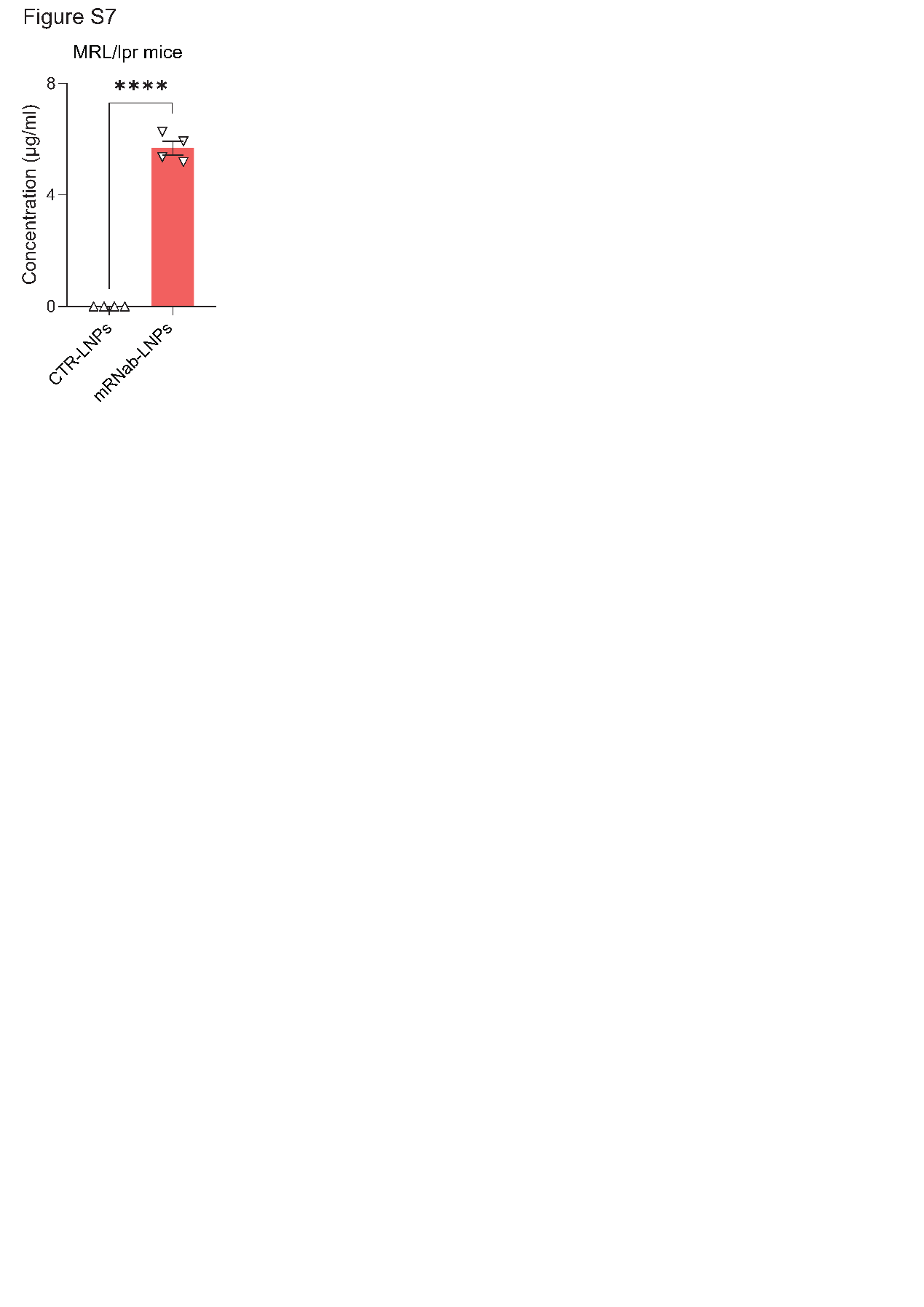

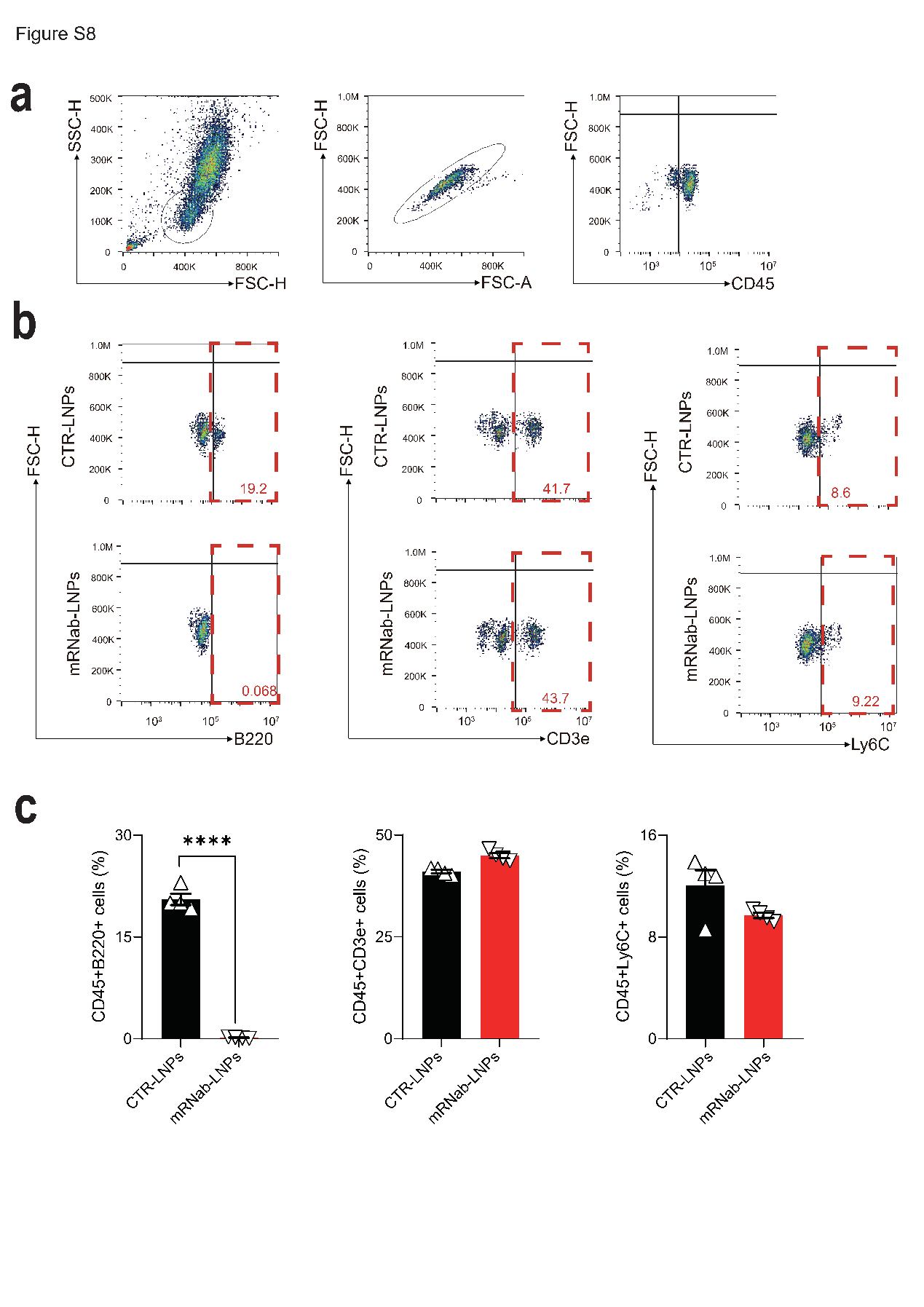

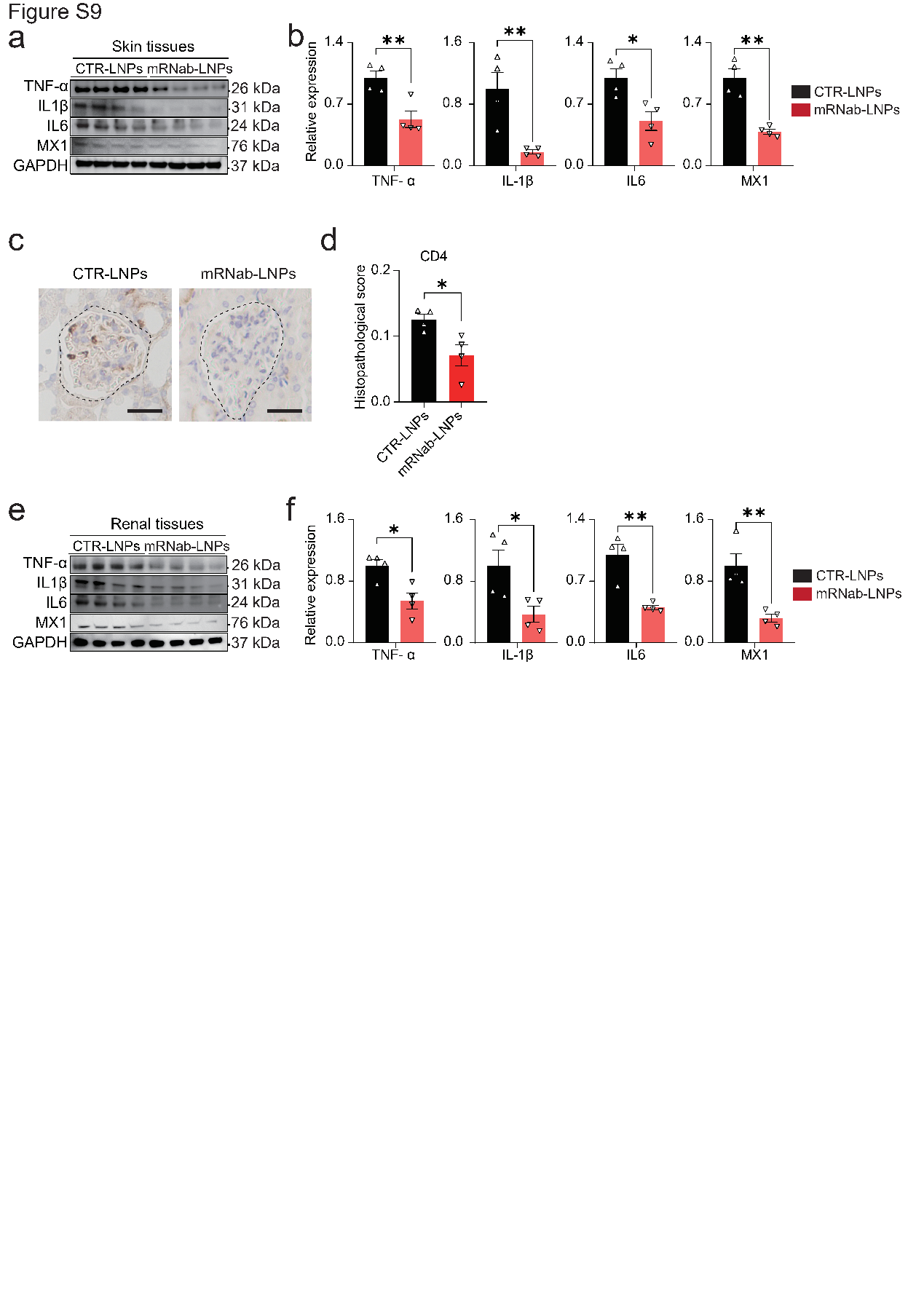

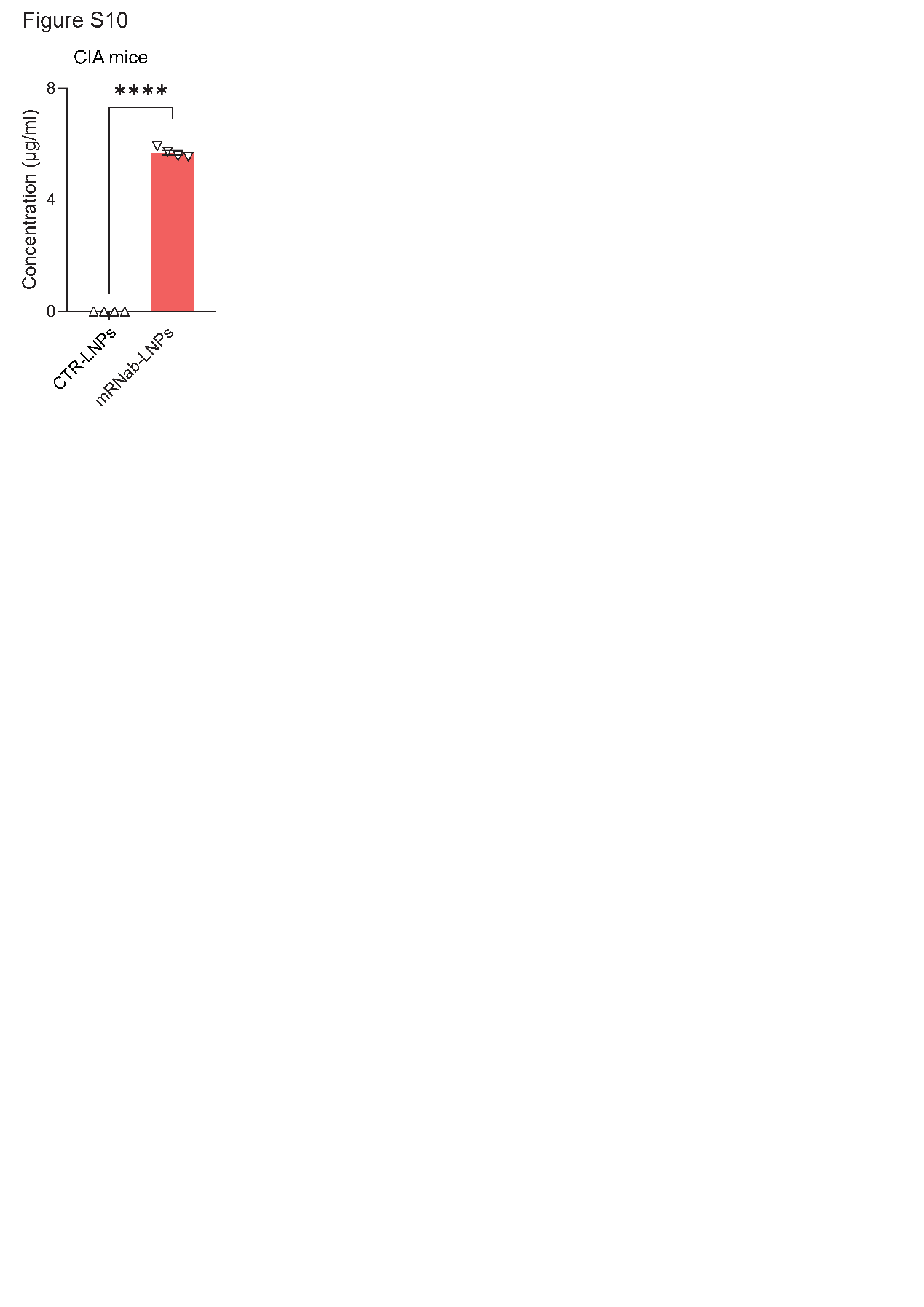

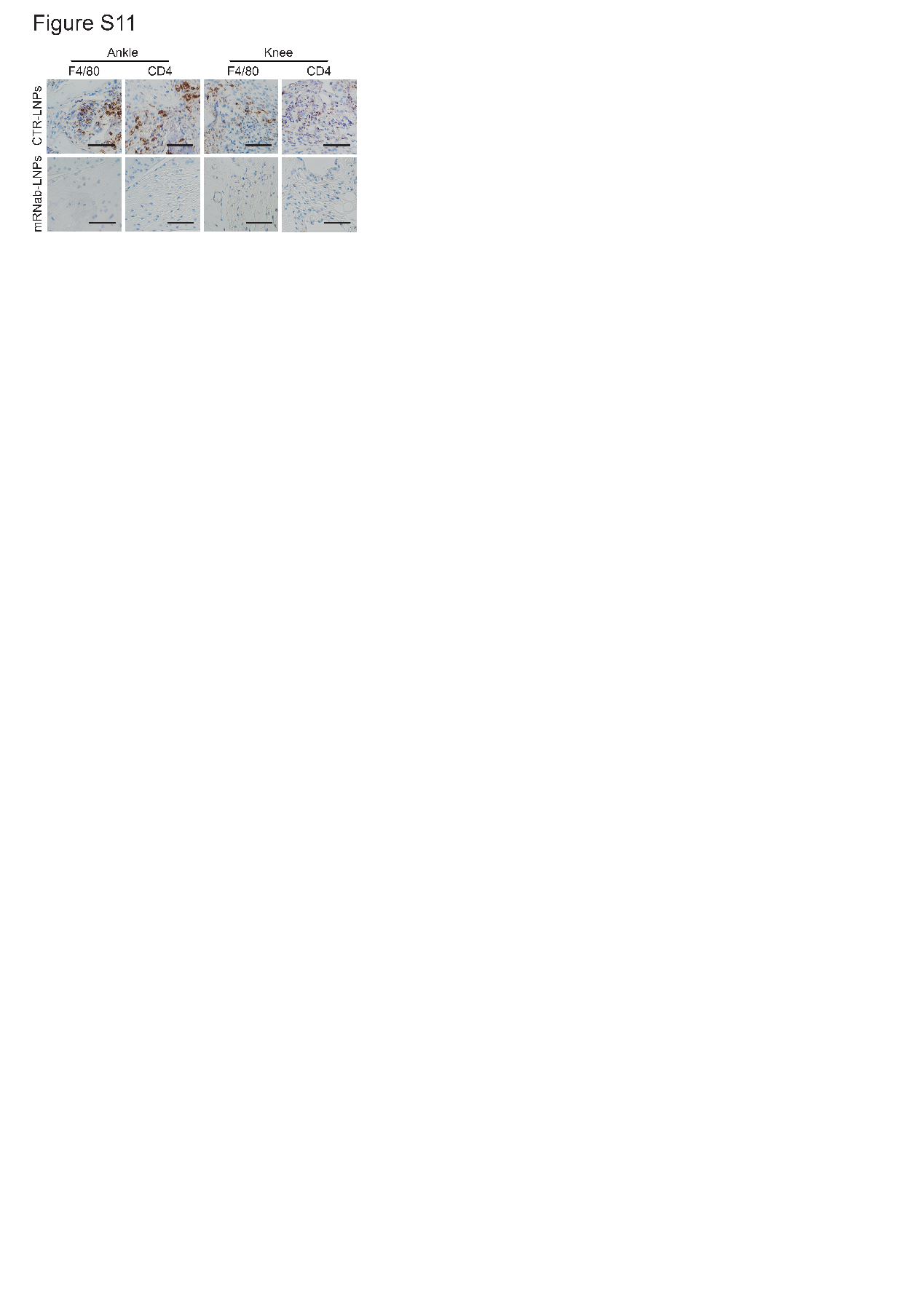

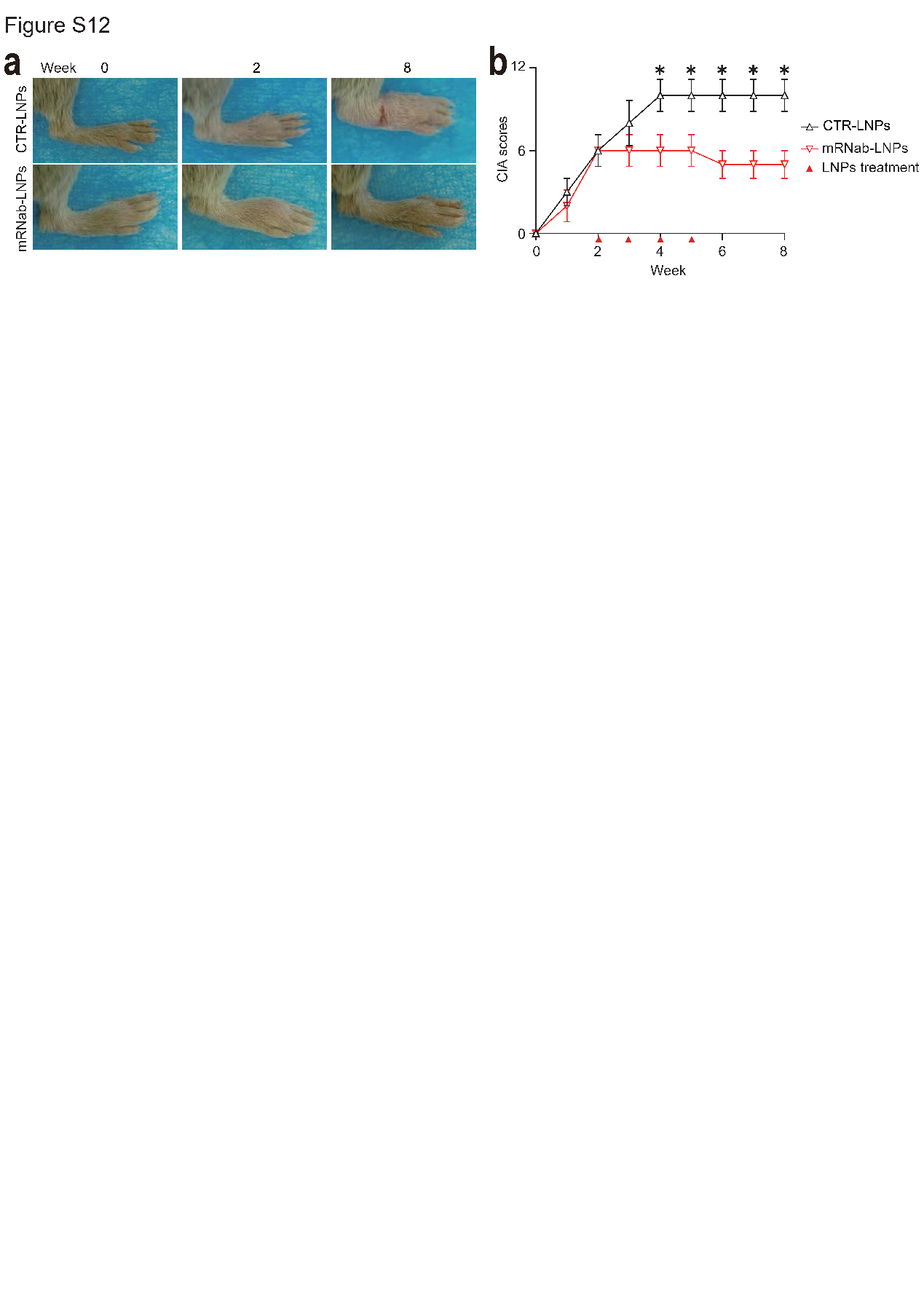

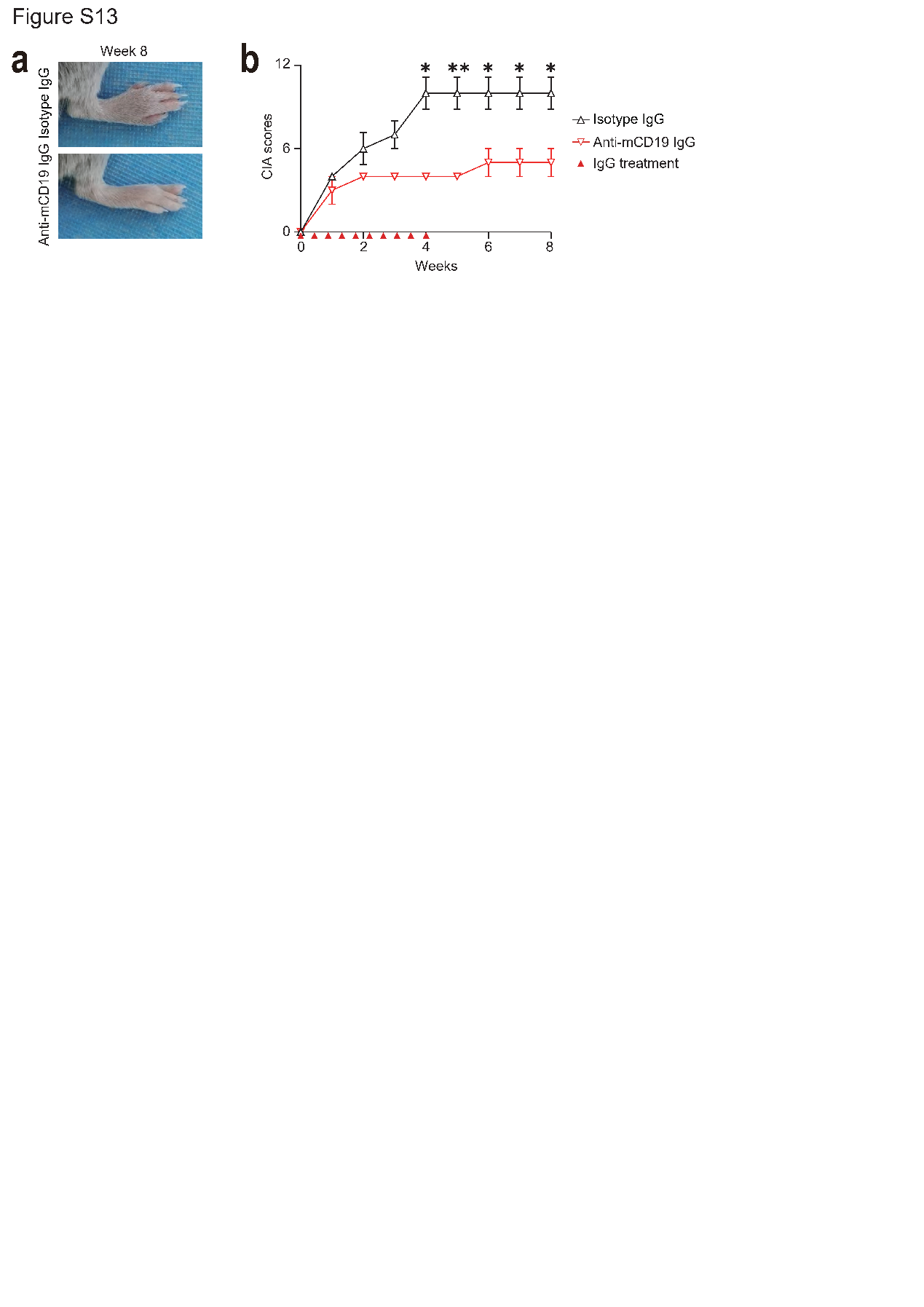

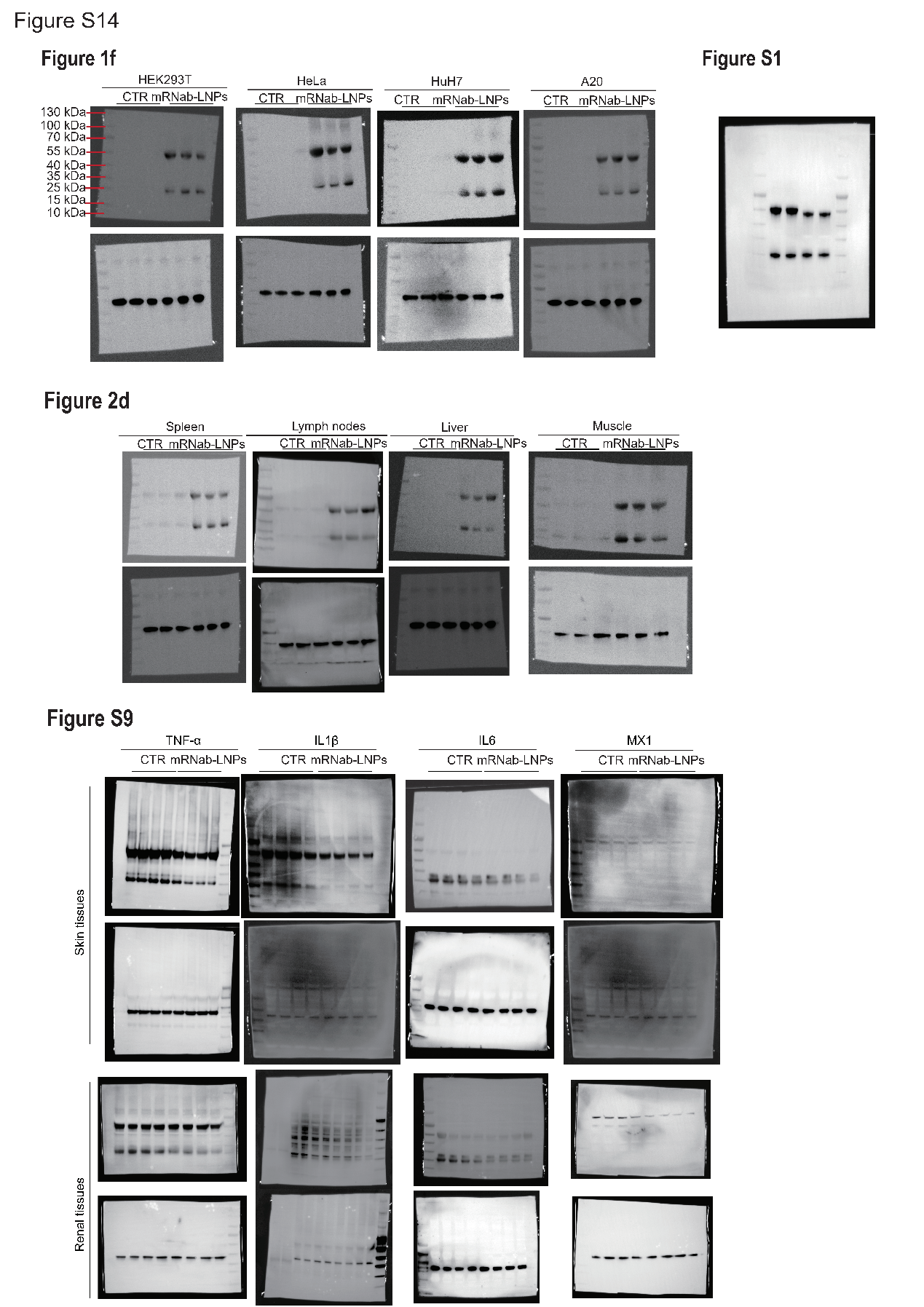

Supplement: Supplementary file 1 — Supporting Information [file ADVS-12-2501628-s001.docx]
